# The effect of polyploidy and hybridization on the evolution of floral colour in *Nicotiana* (Solanaceae)

**DOI:** 10.1093/aob/mcv048

**Published:** 2015-05-15

**Authors:** Elizabeth W. McCarthy, Sarah E. J. Arnold, Lars Chittka, Steven C. Le Comber, Robert Verity, Steven Dodsworth, Sandra Knapp, Laura J. Kelly, Mark W. Chase, Ian T. Baldwin, Aleš Kovařík, Corinne Mhiri, Lin Taylor, Andrew R. Leitch

**Affiliations:** ^1^School of Biological and Chemical Sciences, Queen Mary University of London, Mile End Road, London E1 4NS, UK, ^2^Natural History Museum, London SW7 5BD, UK, ^3^Jodrell Laboratory, Royal Botanic Gardens, Kew, Richmond, Surrey TW9 3DS, UK, ^4^Max Planck Institute for Chemical Ecology, Department of Molecular Ecology, Beutenberg Campus, Hans-Knöll-Strasse 8, 07745 Jena, Germany, ^5^Institute of Biophysics, Academy of Sciences of the Czech Republic, CZ-61265 Brno, Czech Republic, ^6^Institut Jean-Pierre Bourgin, UMR1318 INRA-AgroParisTech, INRA-Versailles, 78026 Versailles cedex, France and ^7^Department of Plant Sciences, University of Cambridge, Downing Street, Cambridge CB2 3EA, UK

**Keywords:** Evolution, floral colour, hybridization, *Nicotiana*, flower pigmentation, pollinator shifts, polyploidy, Solanaceae, spectral reflectance, transgressive traits

## Abstract

**Background and Aims **Speciation in angiosperms can be accompanied by changes in floral colour that may influence pollinator preference and reproductive isolation. This study investigates whether changes in floral colour can accompany polyploid and homoploid hybridization, important processes in angiosperm evolution.

**Methods** Spectral reflectance of corolla tissue was examined for 60 *Nicotiana* (Solanaceae) accessions (41 taxa) based on spectral shape (corresponding to pigmentation) as well as bee and hummingbird colour perception in order to assess patterns of floral colour evolution. Polyploid and homoploid hybrid spectra were compared with those of their progenitors to evaluate whether hybridization has resulted in floral colour shifts.

**Key Results** Floral colour categories in *Nicotiana* seem to have arisen multiple times independently during the evolution of the genus. Most younger polyploids displayed an unexpected floral colour, considering those of their progenitors, in the colour perception of at least one pollinator type, whereas older polyploids tended to resemble one or both of their progenitors.

**Conclusions** Floral colour evolution in *Nicotiana* is weakly constrained by phylogeny, and colour shifts do occur in association with both polyploid and homoploid hybrid divergence. Transgressive floral colour in *N. tabacum* has arisen by inheritance of anthocyanin pigmentation from its paternal progenitor while having a plastid phenotype like its maternal progenitor. Potentially, floral colour evolution has been driven by, or resulted in, pollinator shifts. However, those polyploids that are not sympatric (on a regional scale) with their progenitor lineages are typically not divergent in floral colour from them, perhaps because of a lack of competition for pollinators.

## INTRODUCTION

Polyploidy, or whole-genome multiplication, has played an important role in the evolution of flowering plants (Soltis* et al.*, 2009, 2014). Allopolyploidy, arising from interspecific hybridization and polyploidy, can cause ‘genomic shock’ (McClintock, 1984), which may trigger a suite of genetic changes, including (retro)transposition, differential gene expression, chromosome rearrangements and epigenetic changes (Leitch and Leitch, 2008). These events and novel *cis–trans* interactions between progenitor genomes may generate variation, including transgressive phenotypes, and facilitate rapid divergence of both homoploid and allopolyploid hybrids (Wittkopp* et al.*, 2004; Chen, 2007; Gaeta* et al.*, 2007; Anssour* et al.*, 2009; Tirosh* et al.*, 2009; Clare* et al.*, 2013).

Speciation in angiosperms can be accompanied by, or perhaps driven by, changes in floral colour that may influence pollinator preference and reproductive isolation. Many pollinators, such as bumblebees and hummingbirds, visit a range of flower colours (Waser* et al.*, 1996). Several species of flower-naive bumblebees have an innate colour preference for violet and blue shades, although preferences in experienced foragers are largely determined by learned associations between colours and rewards (Raine* et al.*, 2006). Hummingbirds appear to have no innate preferences for particular colours, but are likewise good at forming associations between flower visual displays and rewards (Goldsmith and Goldsmith, 1979; Chittka and Waser, 1997). Hummingbirds have red receptors, whereas many insects do not. Consequently, red flowers are poorly detectable by bee pollinators, but conspicuous for hummingbirds. Therefore, hummingbird-visited flowers are often red, whereas those pollinated by bees typically have a range of other colours (Rodriguez-Girones and Santamaria, 2004; Shrestha* et al.*, 2013). Flowers visited by nocturnal pollinators are more often white than those pollinated in full daylight, probably to maximize their detectability in dim light conditions (Kevan* et al.*, 1996). Because of such differences in affinities of various pollinator classes to certain flower colours, differences in flower colour can contribute to restricting gene flow between phenotypes, although they will rarely result in complete reproductive isolation; for this, differences in multiple traits are typically essential. In *Aquilegia* (Ranunculaceae), blue-, red- and white/yellow-flowered species are primarily pollinated by bees, hummingbirds and hawkmoths, respectively (Grant, 1952; Whittall and Hodges, 2007). In *Petunia axillaris *(Solanaceae), hawkmoths prefer white flowers to pink flowers transformed to express *ANTHOCYANIN2*, whereas bumblebees prefer pink *ANTHOCYANIN2* flowers to white flowers, demonstrating that expression of a single gene can cause differences in pollinator visitation (Hoballah* et al.*, 2007). Similarly, manipulation of a single locus controlling carotenoid production in *Mimulus* flowers (Phrymaceae) results in a pollinator shift, reaffirming the importance of floral colour in determining pollinator behaviour (Bradshaw and Schemske, 2003).

To analyse floral colour in the context of pollination, it is necessary to consider both colour theory and pollinator visual systems. There are several important differences between the colour vision systems of humans and those of various pollinator types. Humans and many insects are trichromatic, having three discrete photoreceptor types; however, humans possess red- (with peak sensitivity (*λ*_max_) near 560 nm), green- (*λ*_max _= 535 nm) and blue-sensitive (*λ*_max _= 420 nm) photoreceptors (Bowmaker and Dartnall, 1980), whereas many insects have ultraviolet- (UV-, *λ*_max_ ∼ 350 nm), blue- (*λ*_max_ ∼ 440 nm) and green-sensitive (*λ*_max_ ∼ 530 nm) receptors (Peitsch* et al.*, 1992; Briscoe and Chittka, 2001; Kelber* et al.*, 2003). Many birds (Bowmaker, 1998) and some butterflies (Kelber, 2001) have tetrachromatic colour vision. In birds, photoreceptors are sensitive to red, green, blue and either violet or UV wavelengths (Hart and Hunt, 2007). Hummingbirds have four single cone types with peak sensitivities in the UV (*λ*_max _= 370 nm), blue (*λ*_max _= 440 nm), blue–green (*λ*_max _= 508 nm) and yellow (*λ*_max _= 560 nm); the sensitivity of the last extends significantly into the red (Herrera* et al.*, 2008). Hummingbirds can learn to distinguish near-UV light (370 nm) from darkness, confirming that they use their UV receptors for colour vision at a behavioural level (Goldsmith, 1980). We will take into account these differences in pollinator perception as we examine floral colour in the genus *Nicotiana *(Solanaceae).

**Fig. 1.**
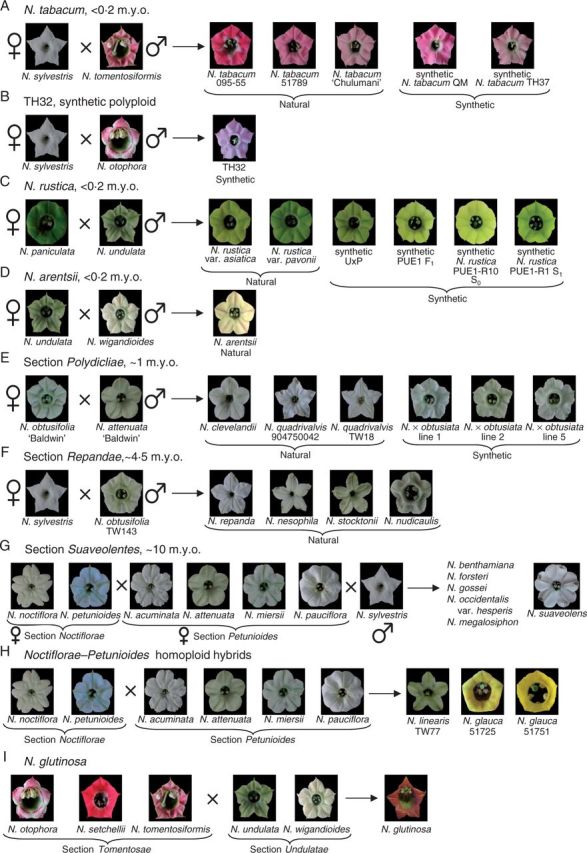
Floral colour, as perceived by humans, of polyploid and homoploid hybrid *Nicotiana* and their diploid progenitors. Polyploid ages were estimated using a molecular clock calibrated with the geological age of volcanic islands with endemic *Nicotiana *species (Clarkson* et al.*, 2005). Absolute dates (millions of years, m.y.o.) estimated by the clock should be treated with caution; however, relative ages of different polyploid sections should reflect the true sequence of polyploidization events. (A) Natural and synthetic polyploids of *N. tabacum*. (B) Synthetic polyploid TH32. (C) Natural and synthetic *N. rustica* polyploids. Synthetic hybrids include a homoploid from a reciprocal cross and a polyploid series (F_1_ homoploid and S_0_ and S_1_ polyploids) of the same parentage as natural *N. rustica*. (D) *Nicotiana arentsii*. (E) Natural polyploids of section *Polydicliae*. Synthetic *N. *× *obtusiata* polyploid lines were made from a cross between the *N. obtusifolia* and *N. attenuata* accessions studied here. (F) Section *Repandae*. (G) Section *Suaveolentes* contains 26 polyploid species (six included in this study). Biogeographical analyses suggest that section *Suaveolentes* originated ∼15 million years ago (m.y.a.), before the aridification of Australia (Ladiges* et al.*, 2011), and this seems to be relatively congruent with the molecular clock results, which place its origin at ∼10 m.y.a. (H) Homoploid hybrids *N. glauca *and *N. linearis*. (I) Homoploid hybrid *N. glutinosa*. Photographs are scaled to the same size.

We investigate the evolution of floral colour across *Nicotiana* (Solanaceae) in the context of polyploidy and hybridization. *Nicotiana* is an excellent group in which to study the effects of hybridization as nearly half of the 76 species are allotetraploids of different ages (Chase* et al.*, 2003; Clarkson* et al.*, 2004, 2005; Leitch* et al.*, 2008; Kelly* et al.*, 2013), and several putative homoploid (diploid) hybrids have also been detected (Clarkson* et al.*, 2010; Kelly* et al.*, 2010), which add to the reticulate nature of the genus. These phylogenetic studies have also been used to predict the closest living descendent species of the parents that formed the homoploid hybrid and allopolyploid species, hereafter called progenitor species, as shown in Fig. 1. Some synthetic polyploids made from these progenitor species are also available, providing insight into the immediate effects of polyploidy and hybridization. We compare floral colours of *Nicotiana* polyploid and homoploid hybrids with those of their diploid progenitors.

Because various animal groups have different sensitivities to colour, it is necessary to model colour perception of specific pollinator classes to understand the significance of floral colour signals. Here, we consider floral colours from a bee perspective (Chittka, 1992), which can also be used to represent other trichromatic insects, such as hawkmoths, due to similarities in photoreceptor sensitivities (Kelber* et al.*, 2003), and a hummingbird perspective (Herrera* et al.*, 2008; Restrepo, 2013). Hummingbirds and hawkmoths are known to visit *Nicotiana* species (Aigner and Scott, 2002; Kaczorowski* et al.*, 2005; Kessler and Baldwin, 2006; Nattero and Cocucci, 2007).

Pigments typically determine floral colour; however, few studies have examined the specific pigments present in *Nicotiana* petals. Aharoni* et al.* (2001) confirm the presence of anthocyanin pigmentation in *N. tabacum*, which seems to be predominantly cyanidin derivatives. Spectral colour shifts can occur in anthocyanins due to hydroxylation and methylation, which result in different types of anthocyanins (Castaneda-Ovando* et al.*, 2009; Andersen and Jordheim, 2010), and differences in pH as well as copigmentation with other pigments, including carotenoids and colourless flavonoids, or metal ions, can also cause spectral shifts in the same anthocyanin compound (Grotewold, 2006; Andersen and Jordheim, 2010). The yellow flower colour of *Nicotiana glauca* is due to carotenoid pigmentation (Zhu* et al.*, 2007). Crossing experiments between diploid *Nicotiana* species suggest that the presence of chlorophyll in corolla tissue is dominant (Brieger, 1935), and similar results corroborate this in the carnation *Dianthus caryophyllus* (Caryophyllaceae; Ohmiya* et al.*, 2014).

In this paper, we seek to determine what types of spectral reflectance are found within *Nicotiana*, and how they appear to bee and hummingbird pollinators. We focus on the consequences of polyploidy and interspecific hybridization on floral colour evolution. Specifically, we aimed to test the hypotheses that: (1) polyploid and homoploid hybrids will have floral colours that will resemble at least one of their progenitors in discrete spectral, bee and hummingbird floral colour categories obtained from cluster analyses; (2) polyploid and homoploid hybrids will be positive for chlorophyll pigmentation in corolla tissue if at least one progenitor has chlorophyll present in its petals (due to evidence of the dominance of chlorophyll pigmentation); (3) increased cell size potentially associated with polyploidy affects the concentration of pigments and, in turn, colour intensity; and (4) floral colour evolution is constrained by phylogeny.

## MATERIALS AND METHODS

### Petal cell area measurements

To assess whether an increase in ploidy results in larger petal cells, cell area was measured from a subset of polyploids and their progenitors. For *Nicotiana sylvestris* A04750326, *Nicotiana rustica* var. *asiatica*, *Nicotiana rustica* var. *pavonii*, *Nicotiana paniculata*, *Nicotiana undulata* and *Nicotiana nudicaulis*, mature flowers were taken from plants and the adaxial petal surface was imprinted in Elite HD vinylpolysiloxane impression material (dental wax, supplied by Zhermack, Harrogate, UK). The wax was left to set, and then used as a mould for making epoxy petal casts. Devcon high-strength epoxy was mixed according to the manufacturer’s instructions, poured into the mould and allowed to set for 12 h. The epoxy relief was removed and coated with gold using a Quorum K756X sputter coater. The samples were then imaged using a FEI Philips XL_30_ FEGSEM scanning electron microscope. For *Nicotiana* *obtusifolia* var. *obtusifolia* TW143, *Nicotiana repanda* and *Nicotiana stocktonii*, only fixed material was available; whole mature flowers were fixed in formalin–acetic acid–alcohol (FAA) (60 % ethanol; 6 % formaldehyde; 5 % acetic acid) for 72 h before being transferred to a 70 % ethanol (EtOH) wash for 24 h. The samples were then dehydrated through an ethanol series of 2 h each in 70, 80 and 90 % and two washes in 100 % EtOH. The samples were dissected and then dried in an Autosamdri 815B critical point dryer. These samples were sputter-coated and imaged as described above. For all samples, images were taken mid-petal from an angle perpendicular to the surface, to minimize parallax error. Cell size measurements were carried out in ImageJ (http://imagej.nih.gov/ij). The circumference of the cell base was drawn freehand and area was calculated for *∼*100–150 cells until the cumulative mean stabilized. One-way ANOVA and Tukey’s honest significance tests were performed in RStudio version 0.98.490 (http://www.rstudio.org) to compare cell area of polyploids with those of their progenitors, repeating the tests for each polyploid section.

### Spectral reflectance measurements

Spectral reflectance measurements were recorded for 60 *Nicotiana* accessions (41 taxa; Supplementary Data Table S1); three flowers from different plants, where possible, were used for each accession. Reflectance spectra from three *Nicotiana otophora* accessions were pooled because the spectra were similar.

Spectral reflectance of flowers at anthesis was measured from 300 to 700 nm using an Avantes AvaSpec-2048 spectrophotometer with an Avantes AvaLight-DHS light source and calibrated with a barium sulphate white standard from labsphere®. *Nicotiana mutabilis* was also measured later, as flowers change from white to pink when mature; pink flowers are less likely to have a nectar reward, but add to the attraction of the overall floral display, and therefore are still relevant to pollinators (R. Kaczorowski, University of Haifa, Israel, pers. comm.). Reflectance spectra express the proportion of light reflected by the flower at any given wavelength. Spectra were visualized and exported in increments of 1 nm using the program AvaSoft version 7.0.3 Full (Avantes BV, Eerbeek, The Netherlands) and imported into Excel.

Spectra for each accession or colour morph were averaged and then smoothed three times, using a rolling average over 9 nm. Spectra for all accessions were submitted to the Floral Reflectance Database (FReD; www.reflectance.co.uk; Arnold* et al.*, 2010).

Some spectra had a spike at ∼656 nm, which corresponded to a narrow peak in the light source spectrum, suggesting that the spectra were saturated at ∼656 nm; however, smoothing served to neutralize this spike. Several spectra (*Nicotiana arentsii*, *N. mutabilis*, *Nicotiana suaveolens* and *Nicotiana wigandioides*) included an anomalous reflectance minimum from 475 to 500 nm, which could not be explained by the light source spectrum. Remeasured spectra of *N. arentsii*, *N. suaveolens* and *N. wigandioides* lacked this minimum, but further material of *N. mutabilis* was unavailable, so these spectra were included despite the anomalies.

### Calculation of colour loci in the bee colour hexagon

A reflectance spectrum can be represented as a single point in the bee colour hexagon space (a graphical representation of a bee’s colour visual experience) based on the relative excitation of UV-, blue- and green-sensitive photoreceptor types (Chittka, 1992). Vertices of this hexagon represent theoretical states where one or two photoreceptor types are at maximal excitation whereas at least one receptor type is at zero excitation (e.g. the top vertex of the hexagon corresponds to maximal blue receptor excitation and zero signal from UV and green receptors, whereas the top right vertex corresponds to maximal signal in both blue and green receptors, but no signal in the UV receptor, and so forth; see Supplementary Data Fig. S1). The centre or origin of the hexagon is achromatic. Hue corresponds to angular position around the origin, whereas spectral purity or saturation increases with distance from the origin.

Bee colour hexagon coordinates were calculated for all *Nicotiana* spectra. Illumination was assumed to be sunlight (D65; Wyszecki and Stiles, 1982); the background was represented by an average leaf spectrum (Gumbert* et al.*, 1999). Honeybee photoreceptor spectral sensitivity functions were used to determine bee colour hexagon coordinates; these are similar to bumblebee and hawkmoth photoreceptor sensitivity functions, so the bee colour hexagon can be used to approximate the colour vision of these insects as well (Menzel* et al.*, 1986; Peitsch* et al.*, 1992; Briscoe and Chittka, 2001; Kelber* et al.*, 2003 and references therein; Skorupski* et al.*, 2007). The equations used to determine colour hexagon coordinates are as follows, where *E*_G_, *E*_B_ and *E*_UV_ represent the excitation of the green, blue and UV bee photoreceptors, respectively, elicited by a spectrum (Chittka, 1992):

$$\begin{array}{l} x=\sqrt{\text{3}}/\text{2}(E_{\text{G}}-E_{\text{UV}})\\ y=E_{\text{B}}-0\cdot \text{5}(E_{\text{UV}}+E_{\text{G}}) \end{array}$$

Because the colour loci of *Nicotiana* flowers were mostly close to the centre of the colour space, all colour hexagon displays presented are scaled so that only the central 40 % is shown; the outline is therefore drawn as a dashed line. This results in a clearer spread of the colour loci to facilitate visual inspection. For a diagram explaining the colour hexagon, see Supplementary Data Fig. S1.

### Calculation of colour loci in hummingbird colour space

For tetrachromatic hummingbirds, we chose to model flower colours in a 3-D colour opponent space because *n* – 1 colour opponent dimensions are necessary to code the information from *n* colour receptors (Chittka, 1996). The hummingbird colour space can be displayed as a rhombic dodecahedron with 14 vertices (Restrepo, 2013). Like the bee colour hexagon, vertices of the space represent states where one, two or three photoreceptor types are at maximal excitation and at least one receptor type is at zero excitation.

Hummingbird colour space coordinates were calculated for all *Nicotiana* spectra. Illumination was again assumed to be sunlight (D65; Wyszecki and Stiles, 1982) and the background an average leaf spectrum (Gumbert* et al.*, 1999) as was used for the bee colour hexagon. Photoreceptor spectral sensitivity functions from the green-backed firecrown hummingbird (*Sephanoides sephanoides*; Herrera* et al.*, 2008) were used to determine hummingbird colour space coordinates using the following equations (Restrepo, 2013), where ER, EG, EB and EUV represent excitation of red, green, blue and UV hummingbird photoreceptors, respectively, elicited by a spectrum:

$$\begin{array}{l} x=\sqrt{\text{3}/\text{4}}E_{\text{B}}-\sqrt{\text{1}/\text{12}}\;(E_{\text{UV}}+E_{\text{G}}+E_{\text{R}})\\ y=\sqrt{\text{2}/\text{3}}E_{\text{G}}-\sqrt{\text{1}/\text{6}}\;(E_{\text{UV}}+E_{\text{R}})\\ z=\sqrt{\text{1}/\text{2}}\;(E_{\text{UV}}-E_{\text{R}}) \end{array}$$

RStudio was used to make 3D plots of the hummingbird colour space, and ImageJ version 1.48 (http://imagej.nih.gov/ij) was used to create an animation of the *Nicotiana* flower loci in the hummingbird colour space. Again, *Nicotiana* flower colour loci are close to the origin in the hummingbird colour space, so the graphs presented display only the central portion (either 25 or 50 %) of the colour space for clarity. To further facilitate interpretation of these graphs, vertices representing individual excitation of the red, green, blue and UV photoreceptor types, as well as their excitation vectors from the origin, are shown in red, green, blue and black, respectively. Other vertices (representing excitation of two or three photoreceptor types) are shown in grey.

### Cluster analyses

Cluster analyses were used to group spectra based on spectral shape (corresponding to pigmentation) and their position in both bee and hummingbird colour spaces. For spectral colour categories, spectra were normalized to the same integral under the curve in order to compare combinations of pigments, not the concentration of pigments. A distance matrix was calculated from the normalized spectral data in R version 3.0.2 (http://www.R-project.org/) using the dist() function. For the bee and hummingbird colour categories, the input data were the (*x*, *y*) or (*x*, *y*, *z*) coordinates of the spectra in the bee and hummingbird colour spaces, respectively.

Data were first imported into R. The function hclust() was used to perform agglomerative hierarchical clustering based on the average pairwise distances between groups. With this algorithm, the observed points, which are initially all deemed to be distinct, are iteratively assigned to groups until eventually all points belong to the same group. At each step, the average distance between all groups (i.e. the mean distance from all points in group A to all points in group B; if either one of these is a single point then no averaging is needed) is calculated, and the two groups with the smallest average distance are merged. The order in which groups are merged can be used to construct a dendrogram showing the spatial relationship between all data points. We can also look at the distribution of merge distances at each step in the algorithm and use this distribution to estimate how many groups are present in the data. Points at which there is a steep increase in the average between-group distance (‘elbow’ points) highlight the spatial scale(s) at which there is clustering present in the data. By using one of these elbow points as a cutoff in the algorithm, we can arrive at a distance grouping that captures the spatial clustering. It should be noted that the determination of where to draw the threshold in a cluster analysis is arbitrary, but the use of one of these elbow points does yield meaningful clusters. The determination of the specific point from the elbow region to be used to define clusters was further informed by visual inspection of reflectance spectra, as well as distributions of colour loci in the perceptual colour spaces. It should be noted that the dendrograms relate to similarities in spectral reflectance, as well as colour relationships perceived by bees and hummingbirds; they do not show phylogenetic relationships.

### Ancestral state reconstruction

To examine the evolution of colour within a phylogenetic context, ancestral state reconstructions were performed on trees inferred from plastid sequence data. Only species for which floral character data are available were included in these analyses. Because polyploid and homoploid hybrids originate via reticulate evolutionary processes and therefore lack a history of tree-like evolution, ancestral characters were reconstructed using only non-hybrid diploid species. The states observed in hybrid species were then compared with the ancestral state reconstructions. Since sections *Repandae *and *Suaveolentes* have diversified to form several species following polyploidization, characters were reconstructed for these sections separately to examine colour evolution subsequent to their origin. For non-hybrid diploid species, individual gene trees yield some conflicting topologies; nevertheless, key nodes for the purposes of interpreting character evolution in hybrids are recovered in multiple gene trees and are supported by supernetwork analyses (Kelly* et al.*, 2010). Therefore, plastid data from previously published studies are suitable for these analyses.

Previously published sequences (Clarkson* et al.*, 2004) from four plastid regions (*mat*K, *ndh*F, *trn*L-F and *trn*S-G) were aligned separately using PRANK+F (Löytynoja and Goldman, 2008) and then concatenated to create a combined plastid dataset before further optimization by eye in Mesquite version 2.74 (Maddison and Maddison, 2008). For *Nicotiana attenuata*, we used GenBank accessions AB040009 and AY098697 for the *matK *and *trnL-F* regions, respectively (due to likely misidentification of *N. attenuata* material used in Clarkson* et al.*, 2004; see Clarkson* et al.*, 2010); the other two regions were scored as missing data. Phylogenetic reconstruction by Bayesian inference was performed as described in Kelly* et al.* (2013) with the exception that BayesTrees v.1.3 (www.evolution.reading.ac.uk/BayesTrees.html) was used to construct 95 % majority rule consensus trees. For sections *Repandae* and *Suaveolentes*, sequences representing their putative maternal progenitors were included during Bayesian inference to allow rooting of trees but were pruned from the trees prior to ancestral state reconstruction.

Ancestral states for spectral reflectance colour categories and presence/absence of chlorophyll in petals (data in Supplementary Data Table S2) were reconstructed using the parsimony reconstruction method in Mesquite version 2.74, under the unordered states assumption. To account for topological uncertainty, character states were reconstructed over all 36 000 post-burn-in trees using the Trace Character Over Trees option and summarized on the 95 % majority rule consensus tree from the Bayesian analysis. Ancestral states were not calculated for bee or hummingbird colour categories because these are perceptual systems and the same colour category can result from different combinations of pigments (e.g. both human pink and human white flowers, which are positive and negative, respectively, for anthocyanin pigmentation, are both classified as bee blue–green); thus, a single colour category does not necessarily have a shared evolutionary history.

### Estimating expected polyploid and homoploid hybrid floral colour

Polyploid and homoploid hybrid floral colours for each accession were compared with those of their diploid progenitors for the spectral, bee and hummingbird colour categories defined by cluster analyses. Floral colour was classified as ‘expected’ if it fell in the colour category of at least one progenitor, or ‘unexpected’ if it was different from both progenitors. Polyploid and homoploid hybrids were also compared with their diploid progenitors for the presence or absence of chlorophyll in corolla tissue. Chlorophyll absorbs at 675 nm *in vivo* (Haardt and Maske, 1987); therefore, the presence of chlorophyll can be inferred from reflectance spectra if there is a reflectance minimum at 675 nm. The presence of chlorophyll in petal tissue appears to be dominant (Brieger, 1935; Ohmiya* et al.*, 2014). Thus, hybrids were classified as expected if they showed chlorophyll pigmentation and as unexpected if they did not because at least one progenitor possessed chlorophyll in all diploid progenitor combinations. For natural homoploid hybrids and polyploid section *Suaveolentes*, where progenitors can only be defined to *Nicotiana *section level, comparisons were made with reconstructed ancestral characters; thus only spectral colour categories and the presence/absence of chlorophyll were examined.

### Phylogenetic signal in floral traits

In order to statistically test for phylogenetic signal in the phenotypic trait data (spectral reflectance, bee and hummingbird colour perception), we used Mantel tests to examine the correlation between phylogenetic distance and each of the respective continuous multidimensional traits (e.g. Cubo* et al.*, 2005; Muchhala* et al.*, 2014). Analyses were restricted to diploid species, excluding homoploid and polyploid hybrids. Trees were edited in Newick format to include additional tips with zero branch lengths for taxa that are multiple in the trait datasets, either due to colour polymorphism (*N. otophora*) or multiple accessions (*N. sylvestris* and *N. obtusifolia *var. *obtusifolia*).

Statistical analyses were performed in R version 3.1.0. Phenotypic distance matrices were first calculated for the three trait datasets using Euclidean distance, and phylogenetic distance matrices were calculated (1) as genetic distance from the plastid alignment and (2) for each of 36 000 post-burn-in Bayesian trees using cophenetic.phylo(), part of the APE package version 3.1-2 (Paradis* et al.*, 2004). The second Bayesian set of tests was performed in order to account for evolutionary processes such as saturation and to estimate how phylogenetic uncertainty affects the correlation. Mantel tests were performed using Pearson’s product-moment correlation coefficient, with 10 000 permutations of each distance matrix to test for significance; the mean *P* value and its standard deviation were calculated for each set of 36 000 Mantel tests from the Bayesian trees, along with the percentage of trees that gave significant correlations. The function mantel() from the vegan package was used (Oksanen* et al.*, 2013).

## RESULTS

### Petal cell area

**Fig. 2.**
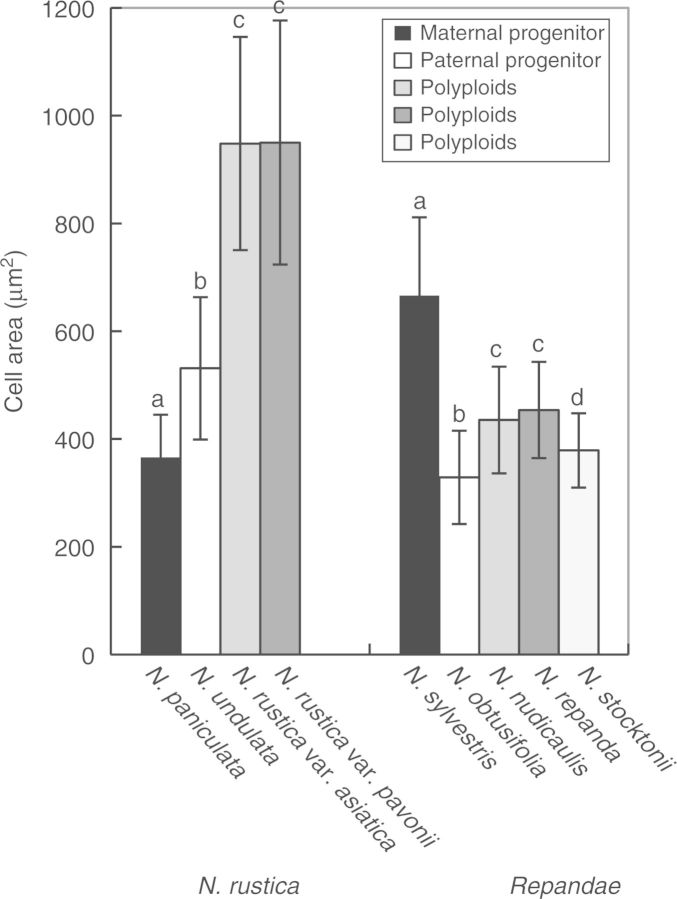
Petal cell area from polyploids and their progenitors. Within each polyploid group, bars with different letters represent significantly different mean cell areas.

Petal cell area was measured to determine whether an increase in ploidy results in larger floral cells. Polyploid petal cell area was significantly larger than in both progenitors in *N. rustica* (ANOVA: *F *= 371, d.f. = 3, *P *< 2 × 10^–^^16^) accessions, but was intermediate between progenitors in section *Repandae* polyploids (ANOVA: *F *= 249·2, d.f. = 4, *P *< 2 × 10^–^^16^; Fig. 2). Accessions that were significantly different in cell area (within polyploid sections and their progenitors) are represented by different letters above the bars in Fig. 2; results from Tukey’s honest significance tests can be found in Supplementary Data Table S3.

### Cluster analyses

**Fig. 3.**
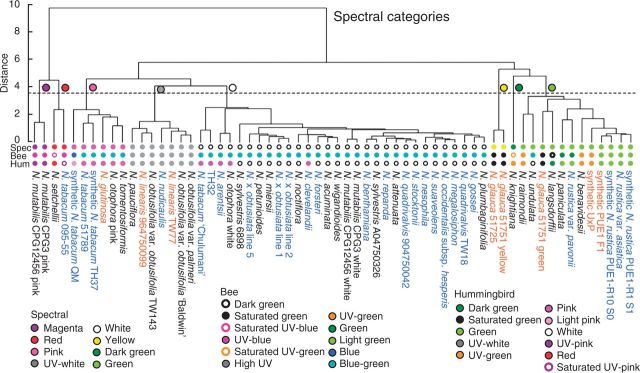
Dendrograms based on distance cluster analyses for spectral reflectance. Coloured circles on the dendrogram represent distinct colour categories as determined by the chosen threshold (dashed line). Dendrograms were similarly constructed for bee and hummingbird colour (see Supplementary Data Fig. S3). The lines of coloured circles at the tips of the dendrograms signify the category each taxon is assigned to in spectral, bee and hummingbird colour as labelled (Spec, Bee and Hum) for comparison between spectral categories and those of different visual systems. Diploids, polyploids and homoploids are denoted by black, blue and orange text, respectively.

**Fig. 4.**
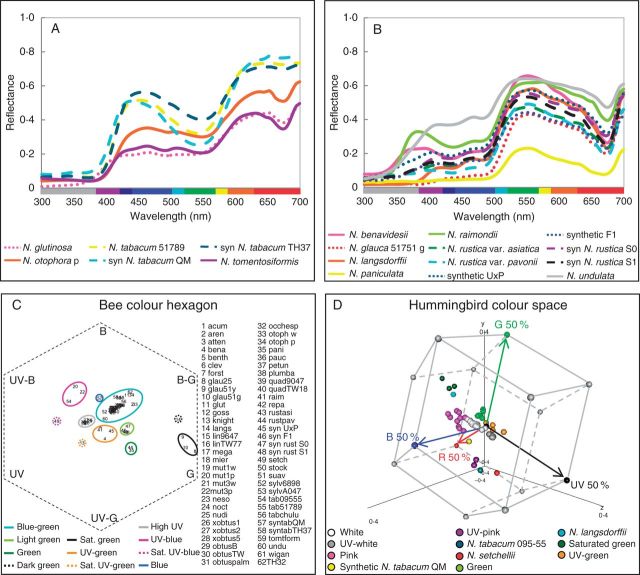
(A, B) *Nicotiana *reflectance spectra from 300 to 700 nm, which roughly correspond to colours perceived by human observers as pink (A) and green (B). See Supplementary Data Fig. S2 for other spectral colour categories. Solid lines are used for diploid taxa, dashed lines for polyploid taxa, and dotted lines for homoploid hybrid taxa. Abbreviations: p, pink; syn, synthetic; g, green. (C) Colour hexagon displaying the distribution of *Nicotiana* colour loci in bee colour space. The hexagon has been scaled so that vertices represent 40 % excitation of photoreceptors. UV, ultraviolet; UV-B, UV–blue; B, blue; B-G, blue–green; G, green; UV-G, UV–green. Bee colour categories are delineated by coloured ovals; sat., saturated. *Nicotiana* species abbreviations are as follows: acum, *acuminata*; aren,* arentsii*; atten,* attenuata*; benavid, *benavidesii*; benth,* benthamiana*; clev,* clevelandii*; forst,* forsteri*; glau25,* glauca *51725; glau51y,* glauca* 51751 yellow; glau51g,* glauca *51751 green; glut,* glutinosa*; goss,* gossei*; knight, *knightiana*; langs,* langsdorffii*; lin9647,* linearis *964750099; linTW77,* linearis *TW77; mega,* megalosiphon*; mier,* miersii*; mut1w,* mutabilis* CPG12456 white; mut1p,* mutabilis* CPG12456 pink; mut3w,* mutabilis* CPG3 white; mut3p,* mutabilis* CPG3 pink; neso,* nesophila*; noct,* noctiflora*; nudi,* nudicaulis*; ×obtus1, × *obtusiata *line 1; ×obtus2, × *obtusiata *line 2; ×obtus5, × *obtusiata *line 5; obtusB,* obtusifolia* var. *obtusifolia *‘Baldwin’; obtusTW,* obtusifolia *var. *obtusifolia* TW143; obtuspalm,* obtusifolia *var.* palmeri*; occhesp, *occidentalis *subsp. *hesperis*; otoph w,* otophora *white; otoph p,* otophora *pink; pani,* paniculata*; pauc,* pauciflora*; petun,* petunioides*; plumba,* plumbaginifolia*; quad9047,* quadrivalvis* 904750042; quadTW18,* quadrivalvis *TW18; raim,* raimondii*; repa,* repanda*; rustasi,* rustica *var.* asiatica*; rustpav,* rustica *var. *pavonii*; syn U×P, synthetic U×P; syn F_1_, synthetic PUE1 F_1_; synrust S0, synthetic* rustica *PUE1-R10 S_0_; synrust S1, synthetic* rustica *PUE1-R1 S_1_; setch,* setchellii*; stock,* stocktonii*; suav,* suaveolens*; sylv6898,* sylvestris *6898; sylvA047,* sylvestris* A04750326; tab09555,* tabacum *095-55; tab51789,* tabacum* 51789; tabchulu,* tabacum* ‘Chulumani’; syntabQM, synthetic* tabacum *QM; syntabTH37, synthetic *tabacum* TH37; tomtform,* tomentosiformis*; undu,* undulata*; wigan,* wigandioides*; TH32, TH32, synthetic *N. sylvestris × N. otophora* polyploid. (D) Distribution of *Nicotiana* spectral loci in hummingbird colour space. Vertices of the hummingbird colour space represent 50 % excitation of the photoreceptors; single photoreceptor type vertices (red, green, blue and UV) are coloured red, green, blue and black, respectively, and all other vertices are grey. Red, green, blue and black arrows represent the vectors of these photoreceptors from the origin of the hummingbird colour space. *Nicotiana* loci are coloured according to hummingbird colour categories (Supplementary Data Fig. S3B), but are labelled with the accession name if the category includes only one taxon. See Supplementary Data Video for an animation of this graph.

*Nicotiana* reflectance spectra were grouped into categories based on spectral shape and position in the bee and hummingbird colour spaces using cluster analyses. Bees and hummingbirds have different photoreceptor sensitivities, and we expect our cluster analyses to reflect these differences in sensory equipment. The analysis based on spectral shape yielded eight colour categories, which roughly corresponded to flowers perceived by human observers as magenta, red, pink, UV–white, white, yellow, green and dark green (Fig. 3). *Nicotiana *spectra are displayed by spectral colour category in Fig. 4A, B, Supplementary Data Fig. S2. The bee colour hexagon clustering resulted in 11 colour categories, which fell into the following areas of bee colour space: saturated green, UV–blue, high UV, UV–green, green, light green, blue–green, dark green, saturated UV–blue, saturated UV–green and blue (the last four categories were each represented by only a single accession; Supplementary Data Fig. S3A). These groups are shown in the bee colour hexagon (Fig. 4C). The hummingbird colour space cluster analysis also produced 11 colour categories: saturated green, green, UV–white, UV–green, pink, white, UV–pink, dark green, light pink, red and saturated UV–pink (again the last four categories include only a single accession; Supplementary Data Fig. S3B). These groups are shown in the hummingbird colour space (Fig. 4D), and the same graph is provided as an animation to better display the 3D nature of the colour space (Supplementary Data Video).

### Evolution of spectral reflectance in polyploids and homoploid hybrids

**Fig. 5.**
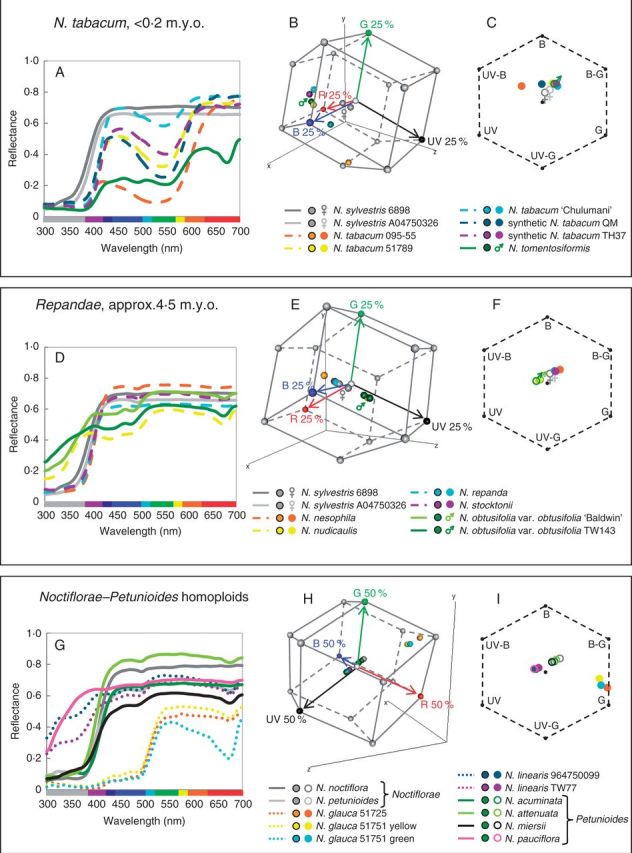
(A, D, G) Reflectance spectra for polyploid and homoploid sections and their progenitors: (A) *N. tabacum*; (D) section *Repandae*; and (G) *Noctiflorae–Petunioides* homoploid hybrids. Solid lines are used for diploid taxa, dashed lines for polyploid taxa and dotted lines for homoploid hybrid taxa. (B, E, H) Hummingbird colour space for polyploid and homoploid sections and their progenitors: (B) *N. tabacum*; (E) section *Repandae*; and (H) *Noctiflorae–Petunioides* homoploid hybrids. The vertices of the hummingbird colour space represent 25 % (B, E) or 50 % (H) excitation of the photoreceptors; single photoreceptor type vertices (red, green, blue and UV) are coloured red, green, blue and black, respectively, and all other vertices are grey. Red, green, blue and black arrows represent the vectors of these photoreceptors from the origin of the hummingbird colour space. (C, F, I) Bee colour hexagons for polyploid or homoploid sections and their progenitors: (C) *N. tabacum*; (F) section *Repandae*; and (I) *Noctiflorae–Petunioides* homoploid hybrids. Hexagons have been scaled so that vertices represent 40 % excitation of photoreceptors. UV, ultraviolet; UV-B, UV–blue; B, blue; B-G, blue–green; G, green; UV-G, UV–green. For information regarding how to interpret colour hexagons, see Supplementary Data Fig. S1. Female (♀) and male (♂) symbols mark maternal and paternal progenitors, respectively, in the hummingbird and bee colour spaces.

To assess the evolution of polyploid floral colour, polyploid and homoploid hybrid accessions were compared with those of their progenitors in spectral, bee and hummingbird colour categories as well as in the presence/absence of chlorophyll. The diploid progenitors and approximate age of polyploids and homoploid hybrids are found in Fig. 1 and Supplementary Data Table S4, and the observed and expected floral colours of polyploid and homoploid hybrids are found in Table 1. Most polyploid and homoploid hybrids were similar to at least one progenitor in spectral, bee and hummingbird colour categories, but some fell into unexpected colour categories (Table 1, Fig. 5, Supplementary Data Fig. S4, Supplementary Data Fig. S5). Over half of the polyploids unexpectedly lacked chlorophyll (Table 1).

**Table 1.** Polyploid and homoploid hybrid observed and expected floral colours

Species	Spectral		Bee		Hummingbird		Chlorophyll	
	Observed	Expected	Observed	Expected	Observed	Expected	Observed	Expected
*N. tabacum *095-55	*R*	W, P	*UV-B*	B-G	*sUV-P*	W, P	*N*	C
*N. tabacum *51789	P	W, P	B-G	B-G	P	W, P	*N*	C
*N. tabacum *‘Chulumani’	W	W, P	B-G	B-G	P	W, P	*N*	C
syn *N. tabacum *QM	P	W, P	*B*	B-G	*LP*	W, P	*N*	C
syn *N. tabacum *TH37	P	W, P	B-G	B-G	P	W, P	*N*	C
TH32	P	W, P	B-G	B-G	P	W, P	*N*	C
*N. rustica *var. *asiatica*	G	G	*LG*	G, B-G	G	G, W	C	C
*N. rustica *var. *pavonii*	G	G	G	G, B-G	G	G, W	C	C
syn U × P (homoploid)	G	G	*UV-G*	G, B-G	*UV-G*	G, W	C	C
syn F_1_ (homoploid)	G	G	*LG*	G, B-G	G	G, W	C	C
syn *N. rustica *S_0_	G	G	*LG*	G, B-G	G	G, W	C	C
syn *N. rustica *S_1_	G	G	*LG*	G, B-G	G	G, W	C	C
*N. arentsii*	W	G, W	B-G	B-G	*P*	W	C	C
*N. clevelandii*	W	UV-W, W	B-G	UV, B-G	*P*	UV-W, W	*N*	C
*N. quadrivalvis *TW18	W	UV-W, W	B-G	UV, B-G	W	UV-W, W	C	C
*N. quadrivalvis *9047	W	UV-W, W	B-G	UV, B-G	W	UV-W, W	C	C
*N. × obtusiata *line 1	W	UV-W, W	B-G	UV, B-G	W	UV-W, W	C	C
*N. × obtusiata *line 2	W	UV-W, W	B-G	UV, B-G	W	UV-W, W	C	C
*N. × obtusiata *line 5	W	UV-W, W	B-G	UV, B-G	W	UV-W, W	C	C
*N. repanda*	W	W, UV-W	B-G	B-G, UV	W	W, UV-W	*N*	C
*N. nesophila*	W	W, UV-W	B-G	B-G, UV	W	W, UV-W	*N*	C
*N. stocktonii*	W	W, UV-W	B-G	B-G, UV	W	W, UV-W	C	C
*N. nudicaulis*	UV-W	W, UV-W	UV	B-G, UV	UV-W	W, UV-W	C	C
*N. benthamiana*	W	W	–	–	–	–	*N*	C
*N. forsteri*	W	W	–	–	–	–	C	C
*N. gossei*	W	W	–	–	–	–	*N*	C
*N. megalosiphon*	W	W	–	–	–	–	*N*	C
*N. occidentalis*	W	W	–	–	–	–	*N*	C
*N. suaveolens*	W	W	–	–	–	–	*N*	C

*N. glauca *51725	*Y*	W	–	–	–	–	C	C
*N. glauca* 51751	*Y,G*	W	–	–	–	–	C	C
*N. linearis *TW77	*UV-W*	W	–	–	–	–	C	C
*N. linearis *9647	*UV-W*	W	–	–	–	–	C	C
*N. glutinosa*	P	P, W, G	–	–	–	–	C	C

The top block consists of polyploid accessions and the bottom block includes homoploid hybrids.

Italic denotes an unexpected phenotype given the colour categories of the progenitors.

Progenitor bee and hummingbird colour categories are unknown for section *Suaveolentes* and natural homoploid hybrids (see text).

R, red; W, white; P, pink; G, green; UV-W, UV–white; Y, yellow; UV-B, UV–blue; B-G, blue–green; B, blue; LG, light green; UV, high UV; UV-G, UV–green; sUV-P, saturated UV–pink; LP, light pink; N, no chlorophyll; C, chlorophyll; syn, synthetic; 9047 represents 904750042; 9647 represents 964750099.

### Evolution of colour characters in a phylogenetic context

**Fig. 6.**
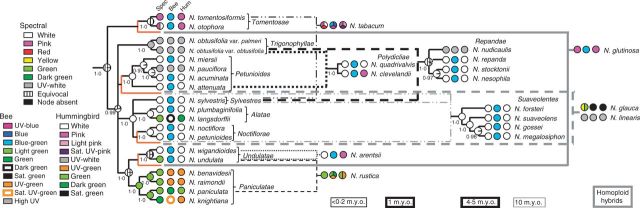
Results of ancestral state reconstruction for spectral colour categories summarized on the 95 % majority rule tree from the Bayesian analysis of plastid sequence data from non-hybrid diploids. Posterior probabilities are shown below the branches. Homoploid and polyploid hybrids are superimposed on the diploid tree; black and grey solid, dashed and dotted lines to the right of the tree represent hybridization events. Orange branches were added to the tree where progenitors of the hybrid taxa are entire sections. Pie charts at internal nodes indicate character states inferred for that node during ancestral state reconstruction carried out on a set of 36 000 post-burn-in trees from the Bayesian analyses. Pie charts at the tips of the branches indicate character states observed in extant species. Bee and hummingbird colour categories for extant species are displayed at the tips of the plastid tree.

Reconstructed character states are shown for spectral reflectance colour categories (Fig. 6) and the presence/absence of chlorophyll in petals (Supplementary Data Fig. S6). Bee and hummingbird colour categories are also shown for extant species on the plastid tree (Fig. 6). Although the deepest nodes were largely equivocal, evolution of spectral reflectance colour in *Nicotiana* seemed to be dynamic (Fig. 6). Green flowers likely have three independent origins: (1) in sections *Paniculatae* and *Undulatae*; (2) in *N. langsdorffii*; and (3) in the homoploid hybrid *N. glauca*. UV–white flowers also seem to have arisen three times independently: (1) in section *Trigonophyllae*; (2) in *N. pauciflora*; and (3) in the homoploid hybrid *N. linearis*. Most polyploid and homoploid hybrid species exhibit a floral colour present in at least one of their progenitors. However, *N. tabacum *095-55 is red and *N. glauca* is yellow and green, unlike their progenitors. UV–white flowers seem to have evolved *de novo* in *N. linearis*. UV–white flowers are also found in one of its progenitor sections (in *N. pauciflora*), but ancestral reconstructions indicate that the floral colour was most likely white at the ancestral nodes within the section (Fig. 6). This suggests that the evolution of UV–white flowers in *N. pauciflora* has occurred subsequent to the formation of *N. linearis* and that the two events are likely independent. It is unclear whether UV–white flowers also evolved *de novo* in *N. nudicaulis* because the ancestral node of section* Repandae *is equivocal. The presence of chlorophyll, as inferred by light absorption at 675 nm (Haardt and Maske, 1987), in *Nicotiana* flowers is ancestral and has been lost three times in *N. sylvestris*, *N. noctiflora* and the most recent common ancestor of *N. acuminata* and *N. pauciflora* (Supplementary Data Fig. S6).

Results from Mantel tests of phylogenetic signal for *Nicotiana* floral traits, for both genetic distance and the 36 000 post-burn-in Bayesian trees, are shown in Table 2. All floral traits were significantly correlated with phylogenetic relationships for the Bayesian trees at a significance level of *P *< 0·05. Only spectral reflectance was significant for the genetic distance tests, whereas bee and hummingbird colour perception were just above the *P *< 0·05 threshold. For the Bayesian trees, 90·1, 66·2 and 93·2 % of trees were significantly correlated with the spectral reflectance, bee and hummingbird colour perception datasets, respectively. These results suggest that these floral traits are weakly constrained by phylogeny.

**Table 2.** Mantel test results

Trait	Genetic distance	Bayesian	
	*P* value	Mean *P* value	% significant trees
Spectral reflectance	0·0229	0·0206 ± 0·0215	90·1
Bee colour vision	0·0866	0·0410 ± 0·0321	66·2
Hummingbird colour vision	0·0594	0·0198 ± 0·0187	93·2

## DISCUSSION

*Nicotiana* is remarkable in its range of spectral reflectance flower colours (white, UV–white, pink, magenta, red, yellow, green and dark green; Fig. 3) and in the variety of pollinators that visit the flowers (moth, bird, bee, bat; Knapp, 2010). The perception of these spectral colours also changes with visual system (bee or hummingbird). Here we describe a complex dynamic in the evolution of floral colour in *Nicotiana*. Spectral reflectance and bee and hummingbird colour perception are correlated with phylogeny, but multiple independent origins of various combinations of pigmentation suggest that the evolution of floral colour is not entirely phylogenetically constrained.

### Petal cell size evolution in polyploids

Cell size is expected to increase following polyploidization due to the increase in genome size (Beaulieu* et al.*, 2008). The significantly larger petal cells of *N. rustica* (<0·2 million years old; m.y.o.) and the intermediate cell size of section *Repandae* (∼4·5 m.y.o.) suggest that polyploids may revert to a diploid-like cell size over time, similar to the genome downsizing observed in polyploids (Leitch and Bennett, 2004). However, petal cell size differences within section *Repandae* do not seem to be linked to genome size; *N. nudicaulis* and *N. repanda* share similar cell sizes, but have substantially different genome sizes (Leitch* et al.*, 2008). The concentration of pigment in floral cells is controlled by both the amount of pigment present and the cell size. *Nicotiana rustica* has significantly larger petal cells than its progenitors (close to the sum; Fig. 2) and displays an intermediate brightness (the area under the reflectance curve, a proxy for pigment concentration) between its progenitors (Supplementary Data Fig. S4D). This is expected if the polyploid inherits the sum of both cell size and amount of pigment present from its progenitors.

### Polyploid divergence in floral colour

Many younger polyploids (<0·2 m.y.o.) display unexpected floral colours, considering those of their diploid progenitors. None of the natural and synthetic *N. tabacum *accessions possess chlorophyll, which is unexpected given its presence in at least one progenitor species. *Nicotiana tabacum *095-55 also has unexpected spectral, bee and hummingbird colour, given the colour categories of the progenitor species. Similarly, synthetic *N. tabacum *QM has unexpected colours in bee and hummingbird perception. Because this accession is synthetic, the parents are known, and thus its unexpected phenotype can be classified as transgressive, or outside the range of its progenitors due solely to polyploidy and hybridization. Most *N. rustica* accessions have unexpected bee colour (four of these are synthetic, and are therefore also transgressive), and *N. arentsii* has unexpected hummingbird colour (Table 1, Fig. 6). Despite the divergence of floral spectra associated with polyploidy, behavioural studies are needed to determine whether the bee and hummingbird colour categories delineated here actually elicit different responses in pollinators.

Most older polyploids (1–10 m.y.o.) are similar in floral colour category to at least one of their progenitors; *N. clevelandii* is the exception because it falls into an unexpected hummingbird colour category, given the progenitor species (Fig. 6). Section *Repandae* polyploids seem to have evolved to be either like their maternal (*Nicotiana nesophila*, *N. repanda *and *N. stocktonii*) or paternal (*N. nudicaulis*) progenitor (Fig. 6). The maternal progenitor, *N. sylvestris*, is no longer sympatric with any of the section *Repandae* polyploids; therefore, *N. nesophila*, *N. repanda* and *N. stocktonii* can occupy the same pollination niche as their maternal progenitor without competition. Similarly, section *Suaveolentes* is native to Australasia, except for one species in Namibia, Africa, and is not sympatric with its progenitor sections in South America (Goodspeed, 1954); these polyploids and their diploid progenitors display similar floral colours, except *N. pauciflora*, which evolved spectrally UV–white flowers after the formation of section *Suaveolentes* (Fig. 6). It is possible that there is less competition for pollinators and, therefore, reduced selective pressure towards floral colour diversification when polyploid species are not sympatric with their diploid progenitors, as is seen in Iochrominae (Solanaceae), which have a broader range of floral colours when species are sympatric (Muchhala* et al.*, 2014). However, floral colour evolution can also be driven by genetic drift or selection on pleiotropic effects of floral genes (Chittka* et al.*, 2001; Rausher, 2008). Furthermore, anthocyanins and flavonoids are important for UV protection, can defend against fungi, act as signal molecules and play a role in male fertility in some species in addition to their roles in signalling to pollinators (Shirley, 1996).

Over half of polyploids have an unexpected inheritance pattern for chlorophyll if it is assumed that the presence of chlorophyll is a dominant character. Those polyploids that deviate from expectation span an age range of synthetic to 10 m.y.o. and always lack chlorophyll in their petals, which is in line with the direction of the shifts observed in the divergence of diploid species (Supplementary Data Fig. S6). In carnation, the difference in chlorophyll concentration between white and green flowers is likely caused by downregulation of chlorophyll biosynthesis genes in white flowers; genes involved in chlorophyll degradation are equally expressed in both flower types (Ohmiya* et al.*, 2014). It is possible that it is advantageous to limit the costs of chlorophyll production when it is unnecessary for photosynthesis, resulting in selection against the presence of chlorophyll in petal tissue. It is also possible that this phenotype results from the silencing of the homeologues that promote chlorophyll biosynthesis.

### Transgressive flower colour in *N. tabacum* and the synthetic polyploid TH32

Polyploids *N. tabacum* and synthetic TH32 are similar because they share a maternal progenitor, *N. sylvestris*, and their paternal progenitors, *Nicotiana tomentosiformis* and *N. otophora*, respectively, are both from section *Tomentosae* and have similar reflectance spectra (Supplementary Data Fig. S5G): the paternal progenitors possess anthocyanin pigmentation as well as chlorophyll, whereas the maternal progenitor lacks both of these.

Genetic crosses in *Nicotiana *suggest that both green flower colour and the ability to produce floral anthocyanins are dominant and each may be determined by a single (likely multigenic) locus (Brieger, 1935). *Nicotiana tabacum* accessions and TH32 possess anthocyanin pigmentation (two spectral peaks in the blue and red portions of the spectrum), but not chlorophyll (the lack of a reflectance minimum at 675 nm) as well as spectral reflectance curve shapes that are distinct from those of their progenitors (Fig. 5A, Supplementary Data Fig. S4A). Therefore, *N. tabacum* and TH32 inherit anthocyanin floral pigmentation from their paternal progenitors, but a plastid phenotype (chlorophyll is only found in plastids) like that of their maternal progenitor, which likely has colourless leucoplasts, as is seen in *Arabidopsis* (Pyke and Page, 1998). Intriguingly, both the *N. tomentosiformis* and *N. sylvestris* copies of the bHLH transcription factor involved in regulation of the anthocyanin biosynthetic pathway are expressed and functional in *N. tabacum* (Bai* et al.*, 2011), suggesting that a maternal gene has been co-opted into producing a paternal-type phenotype.

Polyploids typically inherit plastids from their maternal progenitor; it may be unsurprising, therefore, that *N. tabacum* and TH32 plastids have the maternal phenotype. However, it is likely that the chloroplast-to-leucoplast transition in petal development is regulated by nuclear genes. A study in *Arabidopsis* indicated that petal homeotic genes *APETALA3* and *PISTILLATA* downregulate *BANQUO* genes, which are involved in accumulation of chlorophyll, suggesting that the breakdown of chloroplasts in petal development is linked to repression of genes involved in chlorophyll biosynthesis by nuclear-encoded petal identity genes (Mara* et al.*, 2010). Crosses in carnation and *Nicotiana* provide evidence that maternal plastid phenotype does not determine that of its offspring (Brieger, 1935; Ohmiya* et al.*, 2014), affirming that the plastid phenotype seen in *N. tabacum *and TH32 polyploids is unexpected.

Because this floral phenotype is unlike either progenitor and divergent from the expected phenotype (i.e. the presence of both chlorophyll and anthocyanin pigments from their paternal progenitor) and because the phenotype is seen in synthetic polyploids, it can be considered to be caused by polyploidy and hybridization, and is thus a transgressive phenotype. Because all three natural *N. tabacum* accessions examined show the same phenotype as the synthetic polyploids, we can infer that this unexpected floral phenotype is also transgressive in *N. tabacum*. The observation of this phenotype in at least four independent origins (three synthetic and the natural accessions) suggests that the interplay between the inheritance of plastid and vacuolar pigments yields a transgressive phenotype repeatedly in *N. tabacum* and TH32 polyploids.

*Nicotiana tabacum* varies in spectral shape and bee and hummingbird colour categories among the accessions examined here (Fig. 5A–C). Synthetic *N. tabacum *QM and *N. tabacum *095-55 are unexpected in both bee and hummingbird colour categories, suggesting that these accessions will be distinguishable from their progenitors by both bee (and likely hawkmoth, due to similarities in photoreceptor sensitivities) and hummingbird pollinators. The differences seen among the *N. tabacum* spectra may be due to the presence of different cyanidin derivatives, but vacuolar pH and the formation of heterodimers of anthocyanin and flavonol pigments can also cause shifts in spectral reflectance (Grotewold, 2006; Andersen and Jordheim, 2010).

### Novel floral colour in homoploid hybrids

Over half of the homoploid hybrids examined show unexpected phenotypes in spectral colour categories. Without reproductive isolation, homoploid hybrids often facilitate gene flow between their progenitors instead of becoming established as new species (Buerkle* et al.*, 2000, 2003). In experimental field plots of *Nicotiana alata *and *Nicotiana forgetiana*, pollinator fidelity decreased significantly in the presence of F_1_ hybrids, increasing gene flow between the two progenitor species (Ippolito* et al.*, 2004). Homoploid hybrid *N. glauca* displays a novel floral colour in spectral, bee and hummingbird colour categories (Fig. 6). Although it is the combination of the suite of floral traits displayed that will influence what behaviour a pollinator exhibits, this change in floral colour may have played at least some role in the establishment of reproductive isolation between *N. glauca* and its progenitors.

Species of progenitor sections *Noctiflorae* and *Petunioides* mostly have vespertine flowers and many have long corolla tubes (Goodspeed, 1954), which suggests pollination by nocturnal hawkmoths. The only studies examining pollination in any of these species have confirmed that *N. attenuata* is pollinated by nocturnal hawkmoths but is also visited by hummingbirds (Aigner and Scott, 2002; Kessler and Baldwin, 2006). *Nicotiana glauca* is pollinated by hummingbirds in its native range (Nattero and Cocucci, 2007). Selection can still occur in the presence of generalist pollination based on differences in pollinator assemblage (Gomez* et al.*, 2009), so the floral colour shift in *N. glauca*, accompanied by a shift in the predominant pollinator, may have aided reproductive isolation and its establishment as a new species. Evolutionary shifts in characteristics known to affect pollinator preferences often occur together. A shift from insect to hummingbird pollination has occurred twice within *Mimulus* section *Erythranthe *(Phrymaceae), and red flowers, exserted stamens and pistils and reflexed upper petals (characters associated with hummingbird pollination) seem to have evolved at the same points on the phylogenetic tree as the shift in pollination (Beardsley* et al.*, 2003). In addition to a shift to yellow flowers, *N. glauca* has a reduced floral limb, the part of the corolla that opens (associated with hummingbird pollination), compared with many species in its progenitor sections, suggesting the possibility of hummingbird-mediated selection on *N. glauca* floral traits.

### Concluding remarks

Floral colour shifts in polyploid and homoploid hybrids may occur immediately after their formation, perhaps as a consequence of novel *cis*–*trans* interactions between progenitor genomes (Chen, 2007). Using genomic studies to examine plant–pollinator interactions will shed light on the complex interactions involved in successful pollination and pollinator-mediated evolution (Clare* et al.*, 2013). Transgressive and unexpected floral colours may have aided hybrid speciation, but pollination studies of hybrids and their progenitors are needed to make these conclusions. Typically, synthetic and young polyploids (<0·2 m.y.o.) have floral colour that is unexpected considering the colour of their progenitors in the colour perception of at least one pollinator type. Older polyploids (1–10 m.y.o.) tend to have a floral colour similar to at least one progenitor, perhaps due to the fact that the polyploids are no longer sympatric with one or both progenitors and/or because other floral traits were more important in the divergence from their progenitors.

## SUPPLEMENTARY DATA

Supplementary data are available online at www.aob.oxfordjournals.org and consist of the following. Table S1: *Nicotiana* accessions used in the spectral reflectance dataset and in petal cell area measurements. Table S2: floral colour characters for all *Nicotiana* species examined. Table S3: Tukey’s honest significance test results for cell areas. Table S4: polyploid and homoploid hybrid origins. Figure S1: navigating the bee colour hexagon. Figure S2: *Nicotiana* reflectance spectra from 300 to 700 nm by spectral colour category. Figure S3: dendrograms based on distance cluster analyses for bee and hummingbird colour categories. Figure S4: reflectance spectra, bee colour hexagons and hummingbird colour space for TH32, *N. rustica* and *N. arentsii*. Figure S5: reflectance spectra, bee colour hexagons and hummingbird colour space for section *Polydicliae*, section* Suaveolentes* and *N. glutinosa*. Figure S6: ancestral state reconstruction of the presence/absence of chlorophyll in petals. Video: animation of *Nicotiana* spectra in 3-D hummingbird colour space.

Supplementary Data
