# Supplementary material for: The effect of polyploidy and hybridization on the evolution of floral colour in Nicotiana (Solanaceae)
Source: Ann Bot. 2015 May 15;115(7):1117–31. doi: 10.1093/aob/mcv048 (PMC4598364; doi:10.1093/aob/mcv048)
Supplement: Supplementary Data [file supp_115_7_1117__index.html]

The effect of polyploidy and hybridization on the evolution of floral colour in Nicotiana (Solanaceae) — Supplementary Data 

# The effect of polyploidy and hybridization on the evolution of floral colour in *Nicotiana* (Solanaceae)

## Supplementary Data

files

- Supplementary Data - doc file
