# Supplementary material for: The effect of polyploidy and hybridization on the evolution of floral colour in Nicotiana (Solanaceae)
Source: Ann Bot. 2015 May 15;115(7):1117–31. doi: 10.1093/aob/mcv048 (PMC4598364; doi:10.1093/aob/mcv048)
Supplement: Supplementary Data [file supp_mcv048_mcv048_final-corrections-supp-data.doc]

SUPPLEMENTARY DATA

**Table S1. *Nicotiana* accessions used in the spectral reflectance dataset and in petal cell area measurements**

| Species | Section | Ploidy | No. of flowers^a^ | No. of plants |
| --- | --- | --- | --- | --- |
| *N. acuminata* TW2 | *Petunioides* | diploid | 3 | 3 |
| *N. arentsii* TW12 | *Undulatae* | polyploid | 3 | 1 |
| *N. attenuata* ‘Baldwin’ | *Petunioides* | diploid | 3 | 2 |
| *N. benavidesii* 894750181 | *Paniculatae* | diploid | 3 | 2 |
| *N. benthamiana* TW17 | *Suaveolentes* | polyploid | 3 | 3 |
| *N. clevelandii* TW30 | *Polydicliae* | polyploid | 3 | 1 |
| *N. forsteri* NIC 434/76 | *Suaveolentes* | polyploid | 3 | 1 |
| *N. glauca* 51725 | *Noctiflorae-Petunioides* | homoploid | 3 | 3 |
| *N. glauca* 51751 yellow^b^ | *Noctiflorae-Petunioides* | homoploid | 2 | 2 |
| *N. glauca* 51751 green^b^ | *Noctiflorae-Petunioides* | homoploid | 1 | 1 |
| *N. glutinosa* SCR1 1996 | *Tomentosae-Undulatae* | homoploid | 3 | 2 |
| *N. gossei* NIC 426/78 | *Suaveolentes* | polyploid | 1 | 1 |
| *N. knightiana* CPG | *Paniculatae* | diploid | 3 | 3 |
| *N. langsdorffii* CAM | *Alatae* | diploid | 3 | 3 |
| *N. linearis* 964750099 | *Noctiflorae-Petunioides* | homoploid | 3 | 1 |
| *N. linearis* TW77 | *Noctiflorae-Petunioides* | homoploid | 3 | 1 |
| *N. megalosiphon* NIC 26/80 | *Suaveolentes* | polyploid | 3 | 3 |
| *N. miersii* TW85 | *Petunioides* | diploid | 3 | 3 |
| *N. mutabilis* CPG12456 white^b^ | *Alatae* | diploid | 3 | 3 |
| *N. mutabilis* CPG12456 pink^b^ | *Alatae* | diploid | 3 | 3 |
| *N. mutabilis* CPG3 white^b^ | *Alatae* | diploid | 3 | 3 |
| *N. mutabilis* CPG3 pink^b^ | *Alatae* | diploid | 3 | 3 |
| *N. nesophila* 974750097 | *Repandae* | polyploid | 3 | 1 |
| *N. noctiflora* TW88 | *Noctiflorae* | diploid | 3 | 3 |
| *N. nudicaulis* 964750114** | *Repandae* | polyploid | 3 | 3 |
| *N.* × *obtusiata* line 1 ‘Baldwin’ | synthetic *Polydicliae* | polyploid | 3 | 3 |
| *N.* × *obtusiata* line 2 ‘Baldwin’ | synthetic *Polydicliae* | polyploid | 3 | 3 |
| *N.* × *obtusiata* line 5 ‘Baldwin’ | synthetic *Polydicliae* | polyploid | 3 | 3 |
| *N. obtusifolia* var. *obtusifolia* ‘Baldwin’ | *Trigonophyllae* | diploid | 3 | 1 |
| *N. obtusifolia* var. *obtusifolia* TW143** | *Trigonophyllae* | diploid | 3 | 3 |
| *N. obtusifolia* var. *palmeri* TW98 | *Trigonophyllae* | diploid | 3 | 3 |
| *N. occidentalis* subsp*. hesperis* 974750095 | *Suaveolentes* | polyploid | 3 | 1 |
| *N. otophora* 51727 and 51731 white^b^ | *Tomentosae* | diploid | 5 | 3 |
| *N. otophora* 51727, 51731 and 51739 pink^b^ | *Tomentosae* | diploid | 4 | 3 |
| *N. paniculata*** | *Paniculatae* | diploid | 3 | 3 |
| *N. pauciflora* TW104 | *Petunioides* | diploid | 3 | 3 |
| *N. petunioides* TW105 | *Noctiflorae* | diploid | 3 | 3 |
| *N. plumbaginifolia* TW106 | *Alatae* | diploid | 3 | 1 |
| *N. quadrivalvis* 904750042 | *Polydicliae* | polyploid | 3 | 3 |
| *N. quadrivalvis* TW18 | *Polydicliae* | polyploid | 3 | 3 |
| *N. raimondii* TW109 | *Paniculatae* | diploid | 3 | 3 |
| *N. repanda* TW110** | *Repandae* | polyploid | 3 | 3 |
| *N. rustica* var. *asiatica*** | *Rusticae* | polyploid | 3 | 3 |
| *N. rustica* var. *pavonii*** | *Rusticae* | polyploid | 3 | 3 |
| synthetic (U×P) | synthetic *Paniculatae-Undulatae* | homoploid | 3 | 1 |
| synthetic PUE1 F_1_ | synthetic *Paniculatae-Undulatae* | homoploid | 3 | 1 |
| synthetic *N. rustica* PUE1-R10 S_0_ | synthetic *Rusticae* | polyploid | 3 | 1 |
| synthetic *N. rustica* PUE1-R1 S_1_ | synthetic *Rusticae* | polyploid | 3 | 1 |
| *N. setchellii* | *Tomentosae* | diploid | 3 | 3 |
| *N. stocktonii* TW126** | *Repandae* | polyploid | 3 | 3 |
| *N. suaveolens* CAM | *Suaveolentes* | polyploid | 3 | 3 |
| *N. sylvestris* 6898 | *Sylvestres* | diploid | 3 | 3 |
| *N. sylvestris* A04750326** | *Sylvestres* | diploid | 3 | 2 |
| *N. tabacum* 095-55 | *Nicotiana* | polyploid | 3 | 3 |
| *N. tabacum* 51789 | *Nicotiana* | polyploid | 3 | 1 |
| *N. tabacum* 'Chulumani' | *Nicotiana* | polyploid | 3 | 1 |
| synthetic *N. tabacum* QM | synthetic *Nicotiana* | polyploid | 3 | 2 |
| synthetic *N. tabacum* TH37 | synthetic *Nicotiana* | polyploid | 3 | 1 |
| *N. tomentosiformis* BRNO 4103 | *Tomentosae* | diploid | 3 | 2 |
| *N. undulata* TW145** | *Undulatae* | diploid | 3 | ? |
| *N. wigandioides* | *Undulatae* | diploid | 3 | 1 |
| TH32 (*N. sylvestris* × *N. otophora*) | synthetic *Sylvestres-Tomentosae* | polyploid | 3 | 3 |

**Accessions used for cell area measurements. ^a^Represents the number of flowers used in total, not per plant. ^b^Colours are listed for species which are polymorphic for human floral colour. TW and TH accessions come from the United States *Nicotiana* Germplasm Collection. 9-digit **4750*** accessions come from Radboud University, Nijmegen, The Netherlands. ‘Baldwin’ accessions come from the Baldwin lab (Max Planck Institute, Jena, Germany). CAM accessions come from the Cambridge University Botanic Garden, UK. CPG accessions come from the Chelsea Physic Garden, UK. NIC **/** and 095-55 accessions come from IPK Gatersleben, Germany. 517** accessions were collected in Bolivia; the number refers to the field collection numbers of M. Nee (New York Botanical Garden, USA).

**Table S2** Floral colour characters for all *Nicotiana* species examined

| Species | Spectral reflectance colour | Bee colour | Hummingbird colour | Presence of chloroplasts in petals |
| --- | --- | --- | --- | --- |
| *N. acuminata* TW2 | white | blue-green | white | no |
| *N. arentsii* TW12 | white | blue-green | pink | yes |
| *N. attenuata* ‘Baldwin’ | white | blue-green | white | yes |
| *N. benavidesii* 894750181 | green | UV-green | UV-green | yes |
| *N. benthamiana* TW17 | white | blue-green | white | no |
| *N. clevelandii* TW30 | white | blue-green | pink | no |
| *N. forsteri* NIC 434/76 | white | blue-green | white | yes |
| *N. glauca* 51725 | yellow | saturated green | saturated green | yes |
| *N. glauca* 51751 yellow | yellow | saturated green | saturated green | yes |
| *N. glauca* 51751 green | green | saturated green | saturated green | yes |
| *N. glutinosa* SCR1 1996 | pink | blue-green | pink | yes |
| *N. gossei* NIC 426/78 | white | blue-green | white | no |
| *N. knightiana* CPG | dark green | saturated UV-green | UV-green | yes |
| *N. langsdorffii* CAM | green | dark green | dark green | yes |
| *N. linearis* 964750099 | UV-white | high UV | UV-white | yes |
| *N. linearis* TW77 | UV-white | high UV | UV-white | yes |
| *N. megalosiphon* NIC 26/80 | white | blue-green | white | no |
| *N. miersii* TW85 | white | blue-green | white | yes |
| *N. mutabilis* CPG12456 white | white | blue-green | white | no |
| *N. mutabilis* CPG12456 pink | magenta | UV-blue | UV-pink | no |
| *N. mutabilis* CPG3 white | white | blue-green | white | no |
| *N. mutabilis* CPG3 pink | magenta | UV-blue | UV-pink | no |
| *N. nesophila* 974750097 | white | blue-green | white | no |
| *N. noctiflora* TW88 | white | blue-green | white | no |
| *N. nudicaulis* 964750114 | UV-white | high UV | UV-white | yes |
| *N.* × *obtusiata* line 1 ‘Baldwin’ | white | blue-green | white | yes |
| *N.* × *obtusiata* line 2 ‘Baldwin’ | white | blue-green | white | yes |
| *N.* × *obtusiata* line 5 ‘Baldwin’ | white | blue-green | white | yes |
| *N. obtusifolia* var. *obtusifolia* ‘Baldwin’ | UV-white | high UV | UV-white | yes |
| *N. obtusifolia* var. *obtusifolia* TW143 | UV-white | high UV | UV-white | yes |
| *N. obtusifolia* var. *palmeri* TW98 | UV-white | high UV | UV-white | yes |
| *N. occidentalis* subsp. *hesperis* 974750095 | white | blue-green | white | no |
| *N. otophora* 51727 and 51731 white | white | blue-green | pink | yes |
| *N. otophora* 51727, 51731 and 51739 pink | pink | blue-green | pink | yes |
| *N. paniculata* | green | green | green | yes |
| *N. pauciflora* TW104 | UV-white | high UV | UV-white | no |
| *N. petunioides* TW105 | white | blue-green | white | yes |
| *N. plumbaginifolia* TW106 | white | blue-green | white | yes |
| *N. quadrivalvis* 904750042 | white | blue-green | white | yes |
| *N. quadrivalvis* TW18 | white | blue-green | white | yes |
| *N. raimondii* TW109 | green | UV-green | UV-green | yes |
| *N. repanda* TW110 | white | blue-green | white | no |
| *N. rustica* var. *asiatica* | green | light green | green | yes |
| *N. rustica* var. *pavonii* | green | green | green | yes |
| synthetic (U×P) | green | UV-green | UV-green | yes |
| synthetic PUE1 F_1_ | green | light green | green | yes |
| synthetic *N. rustica* PUE1-R10 S_0_ | green | light green | green | yes |
| synthetic *N. rustica* PUE1-R1 S_1_ | green | light green | green | yes |
| *N. setchellii* | red | saturated UV-blue | red | yes |
| *N. stocktonii* TW126 | white | blue-green | white | yes |
| *N. suaveolens* CAM | white | blue-green | white | no |
| *N. sylvestris* 6898 | white | blue-green | white | no |
| *N. sylvestris* A04750326 | white | blue-green | white | no |
| *N. tabacum* 095-55 | red | UV-blue | saturated UV-pink | no |
| *N. tabacum* 51789 | pink | blue-green | pink | no |
| *N. tabacum* 'Chulumani' | white | blue-green | pink | no |
| synthetic *N. tabacum* QM | pink | blue | light pink | no |
| synthetic *N. tabacum* TH37 | pink | blue-green | pink | no |
| *N. tomentosiformis* BRNO 4103 | pink | blue-green | pink | yes |
| *N. undulata* TW145 | green | blue-green | white | yes |
| *N. wigandioides* | white | blue-green | white | yes |
| TH32 (*N. sylvestris* × *N. otophora*) | white | blue-green | pink | no |

**Table S3** Tukey’s Honest Significance Test results for cell areas

| Polyploid section | Species pair | Difference | p-value |
| --- | --- | --- | --- |
| *N. rustica* | *N. rustica* var. *asiatica*-*N. paniculata* | 582.3 | <0.0001 |
|  | *N. rustica* var. *pavonii*-*N. paniculata* | 584.1 | <0.0001 |
|  | *N. undulata*-*N. paniculata* | 165.1 | <0.0001 |
|  | *N. rustica* var. *pavonii*-*N. rustica* var. *asiatica* | 1.8 | 0.9998 |
|  | *N. undulata*-*N. rustica* var. *asiatica* | -417.2 | <0.0001 |
|  | *N. undulata*-*N. rustica* var. *pavonii* | -419.0 | <0.0001 |
| Section *Repandae* | *N. obtusifolia*-*N. nudicaulis* | -106.7 | <0.0001 |
|  | *N. repanda*-*N. nudicaulis* | 18.5 | 0.6359 |
|  | *N. stocktonii*-*N. nudicaulis* | -56.5 | 0.0002 |
|  | *N. sylvestris*-*N. nudicaulis* | 231.0 | <0.0001 |
|  | *N. repanda*-*N. obtusifolia* | 125.2 | <0.0001 |
|  | *N. stocktonii*-*N. obtusifolia* | 50.2 | 0.0004 |
|  | *N. sylvestris*-*N. obtusifolia* | 337.6 | <0.0001 |
|  | *N. stocktonii-N. repanda* | -75.0 | <0.0001 |
|  | *N. sylvestris*-*N. repanda* | 21.5 | <0.0001 |
|  | *N. sylvestris*-*N. stocktonii* | 287.5 | <0.0001 |

**Table S4 Polyploid and homoploid hybrid origins**

| Hybrid | Maternal Progenitor | Paternal Progenitor | Age (millions of years) |
| --- | --- | --- | --- |
| *N. tabacum* | *N. sylvestris* | *N. tomentosiformis* | <0.2 (Clarks*on et a*l., 2005) |
| synthetic *N. tabacum* QM | *N. sylvestris* | *N. tomentosiformis* | 0 (cross by K. Y. Lim, QMUL, UK) |
| synthetic *N. tabacum* TH37 | *N. sylvestris* | *N. tomentosiformis* | 0 (Burk, 1973) |
| TH32 | *N. sylvestris* | *N. otophora* | 0 (United State*s Nicotia*na Germplasm Collection; Mo*on et a*l., 2008) |
| *N. rustica* | *N. paniculata* | *N. undulata* | <0.2 (Clarkson, 2006; Leit*ch et a*l., 2008) |
| synthetic U×P | *N. undulata* | *N. paniculata* | 0 (diploid cross, A. Kovařík) |
| synthetic PUE1 F_1_ | *N. paniculata* | *N. undulata* | 0 (diploid cross, A. Kovařík) |
| synthetic *N. rustica* PUE1-R10 S_0_ | *N. paniculata* | *N. undulata* | 0 (synthetic PUE1 F_1_ doubled, C. Mhiri) |
| synthetic *N. rustica* PUE1-R1 S_1_ | *N. paniculata* | *N. undulata* | 0 (putative S_1_ from doubled PUE1 F_1_) |
| *N. arentsii* | *N. undulata* | *N. wigandioides* | <0.2 (Clarkson, 2006; Leit*ch et a*l., 2008) |
| *N. clevelandii* | *N. obtusifolia* | *N. attenuata* | ~1 (Clarkson, 2006; Leit*ch et a*l., 2008) |
| *N. quadrivalvis* | *N. obtusifolia* | *N. attenuata* | ~1 (Clarkson, 2006; Leit*ch et a*l., 2008) |
| *N.* × *obtusiata* lines 1, 2, and 5 | *N. obtusifolia* ‘Baldwin’ | *N. attenuata* ‘Baldwin’ | 0 (Ansso*ur et a*l., 2009) |
| *N. repanda* | *N. sylvestris* | *N. obtusifolia* | ~4.5 (Clarks*on et a*l., 2005) |
| *N. nesophila* | *N. sylvestris* | *N. obtusifolia* | ~4.5 (Clarks*on et a*l., 2005) |
| *N. stocktonii* | *N. sylvestris* | *N. obtusifolia* | ~4.5 (Clarks*on et a*l., 2005) |
| *N. nudicaulis* | *N. sylvestris* | *N. obtusifolia* | ~4.5 (Clarks*on et a*l., 2005) |
| *N. benthamiana* | sections *Noctiflorae* and *Petunioides* | *N. sylvestris* | ~10 (Leitch et al., 2008) |
| *N. forsteri* | sections *Noctiflorae* and *Petunioides* | *N. sylvestris* | ~10 (Leitch et al., 2008) |
| *N. gossei* | sections *Noctiflorae* and *Petunioides* | *N. sylvestris* | ~10 (Leitch et al., 2008) |
| *N. megalosiphon* | sections *Noctiflorae* and *Petunioides* | *N. sylvestris* | ~10 (Leitch et al., 2008) |
| *N. occidentalis* subsp. *hesperis* | sections *Noctiflorae* and *Petunioides* | *N. sylvestris* | ~10 (Leitch et al., 2008) |
| *N. suaveolens* | sections *Noctiflorae* and *Petunioides* | *N. sylvestris* | ~10 (Leitch et al., 2008) |
| *N. glauca^*^* | Progenitors: sections *Noctiflorae* and *Petunioides* | | N/A |
| *N. linearis^*^* | Progenitors: sections *Noctiflorae* and *Petunioides* | | N/A |
| *N. glutinosa^*^* | Progenitors: sections *Tomentosae* and *Undulatae* | | N/A |

**^*^**Homoploid hybrid evolution is more convoluted and difficult to detect; therefore, which progenitor was maternal or paternal, as well as the age of origin, has not been determined.

**Fig S1.** Navigating the bee colour hexagon. Based on Chittka (1992). Each vertex is labelled based on which photoreceptor types are at maximal excitation at that point: B=blue, B-G=blue-green, G=green, UV-G=UV-green, UV=UV, UV-B=UV-blue. (A) Excitation vectors of the UV, blue, and green bee photoreceptors, E_UV_, E_B_, and E_G_, respectively, which determine the bee colour space. Excitation vectors range from 0 (no excitation) to 1 (maximal excitation) and are independent of each other. (B) 40% excitation hexagon. All colour hexagons presented here are scaled to 40% excitation for clarity because all *Nicotiana* spectra fall within this hexagon. (C) Hue changes as one moves clockwise or anti-clockwise around the origin. (D) Saturation changes as one moves toward or away from the origin. (E) The three excitation vectors elicited by a spectrum determine the locus of the spectrum in the bee colour hexagon space. Here, E_G_=0.6, E_UV_=0.8, and E_B_=0.2.


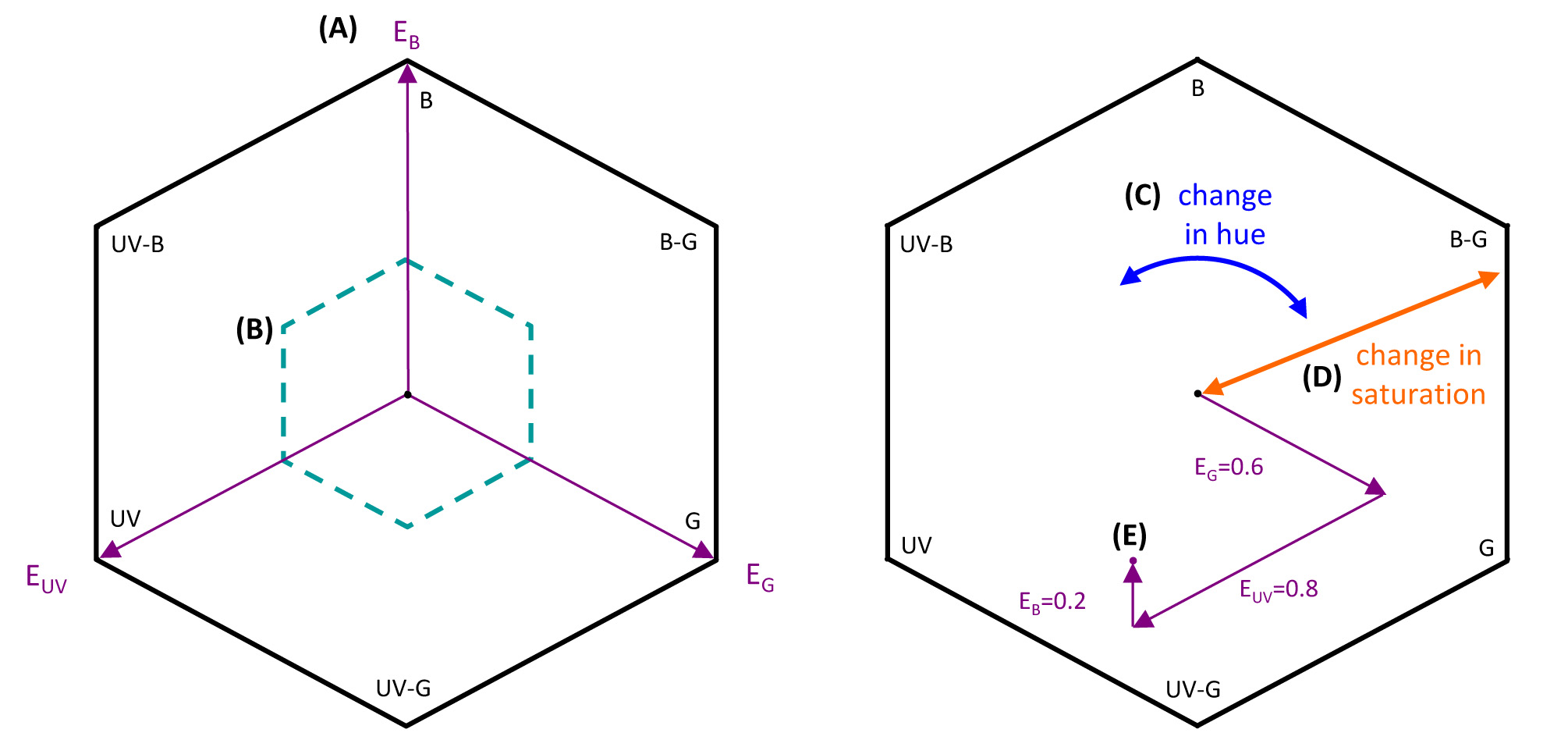


**Fig S2.** *Nicotiana* reflectance spectra from 300-700 nm by spectral colour category, which roughly correspond to colours perceived by human observers as: (A,B) white, (C) UV-white and (D) other categories (magenta, red, yellow and dark green). Solid lines are used for diploid taxa, dashed lines for polyploid taxa, and dotted lines for homoploid hybrid taxa. w=white; 9047=904750042; A047=A04750326; ‘Chulu’=’Chulumani’; 9647=964750099; p=pink; y=yellow. The spectra perceived by humans as white were split into two graphs for clarity.

**
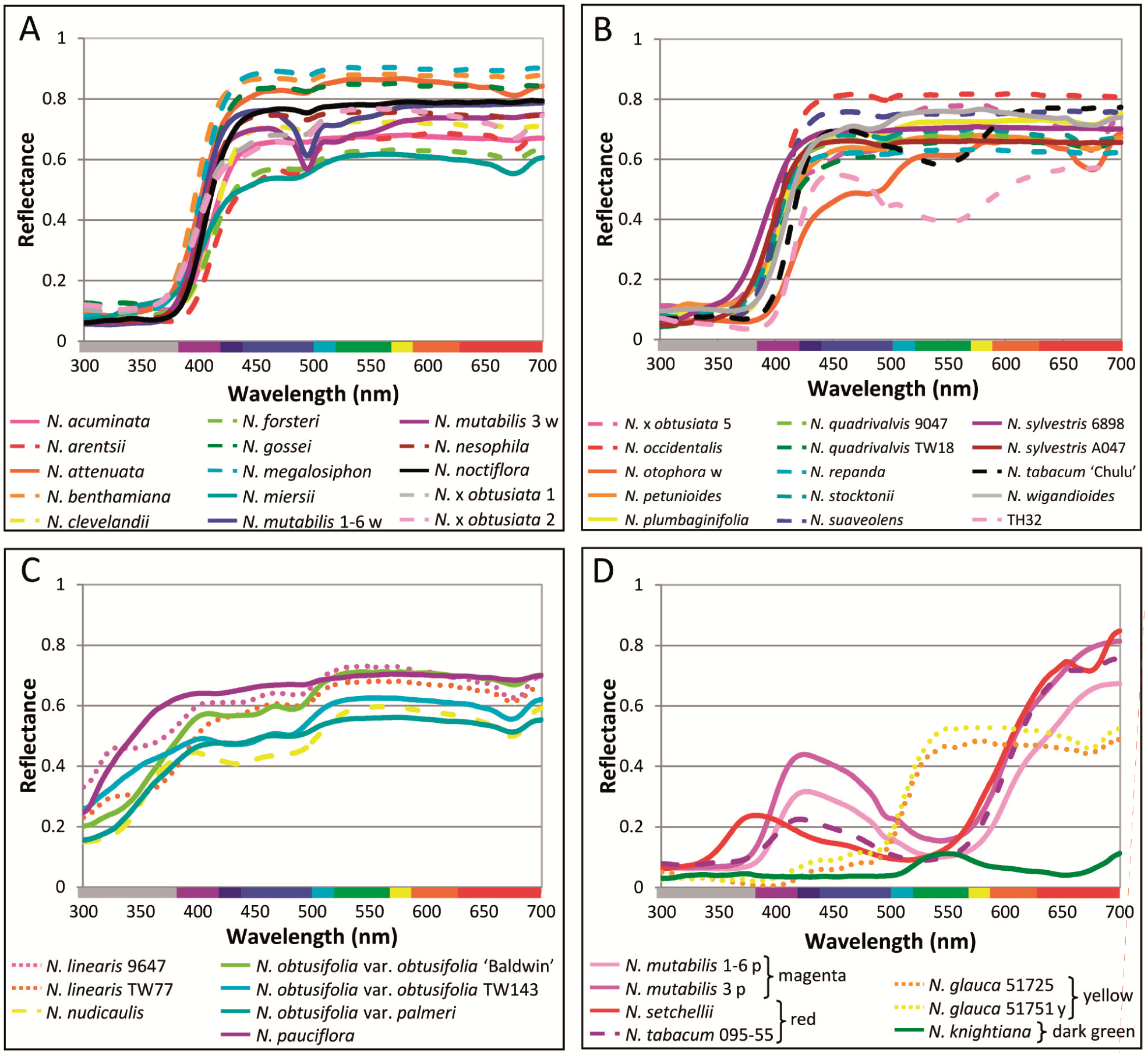
**

**Fig. S3** Dendrograms based on distance clustering for (A) bee and (B) hummingbird colour. Coloured circles on dendrogram represent distinct colour categories as determined by the chosen threshold (dotted line). The lines of coloured circles at the tips of the dendrograms signify the category each taxon is assigned to in spectral, bee and hummingbird colour as labelled (Spec, Bee and Hum) for comparison between spectral categories and those of different visual systems. Diploids, polyploids and homoploids are denoted by black, blue and orange text, respectively.

**
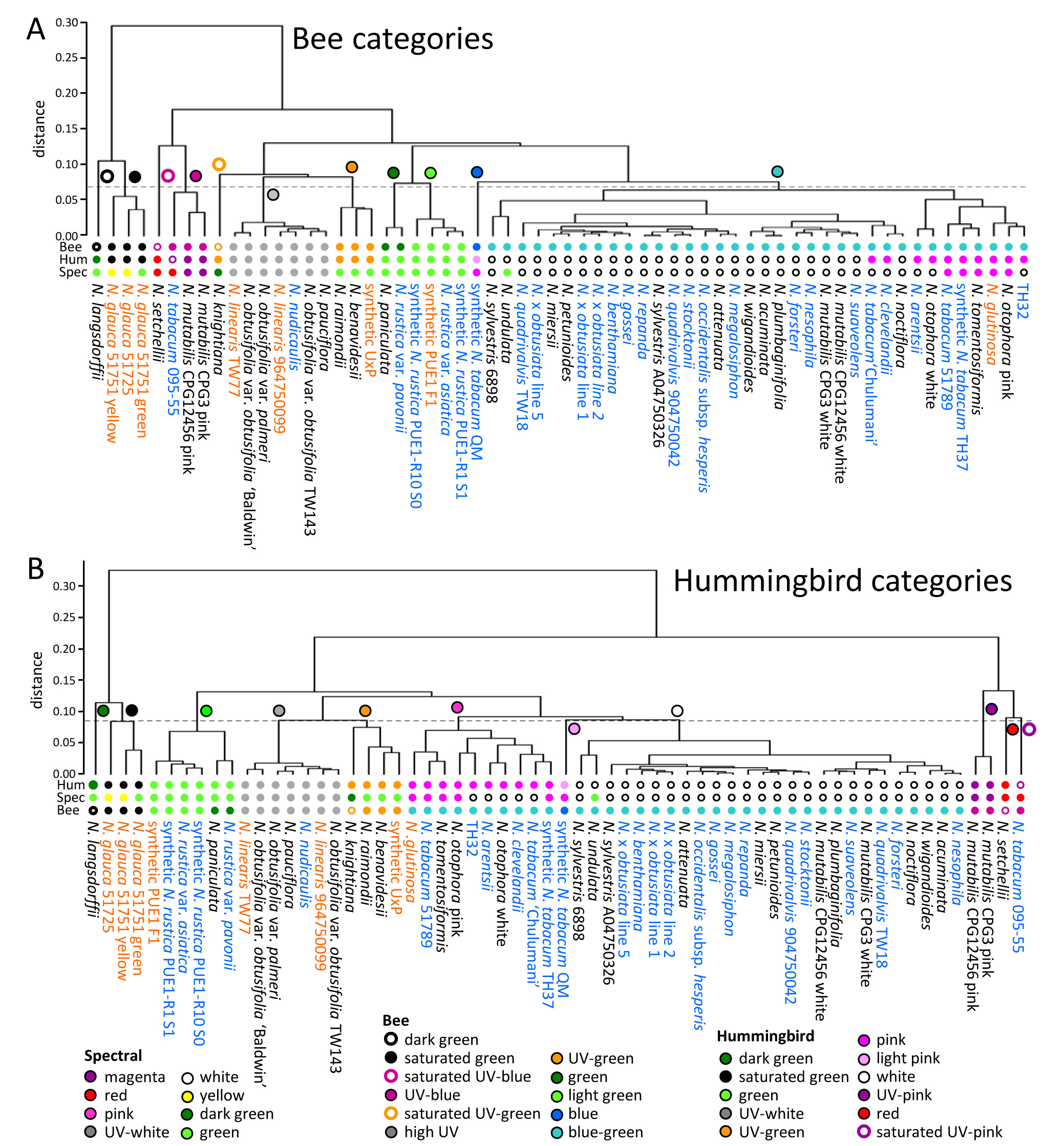
**

**Fig. 4** (A,D,G) Reflectance spectra for polyploid or homoploid sections and their progenitors (A) TH32, (D) *N. rustica* (G) *N. arentsii*. Solid lines are used for diploid taxa, dashed lines for polyploid taxa, and dotted lines for homoploid hybrid taxa. (B,E,H) Hummingbird colour space for polyploid or homoploid sections and their progenitors: (B) TH32, (E) *N. rustica*, (H) *N. arentsii*. The vertices of the hummingbird colour space represent 25% excitation of the photoreceptors; single photoreceptor type vertices (red, green, blue and UV) are coloured red, green, blue and black, respectively and all other vertices are grey. Red, green, blue and black arrows represent the vectors of these photoreceptors from the origin of the hummingbird colour space. (C,F,I) Bee colour hexagons for polyploid or homoploid sections and their progenitors: (C) TH32, (F) *N. rustica*, (I) *N. arentsii*. Hexagons have been scaled so that vertices represent 40% excitation of photoreceptors. UV=ultraviolet; UV-B=UV-blue; B=blue; B-G=blue-green; G=green; UV-G=UV-green. For information regarding how to interpret colour hexagons, see Supplemental Fig. S1. Female (♀) and male (♂) symbols mark maternal and paternal progenitors, respectively, in the hummingbird and bee colour spaces.

**
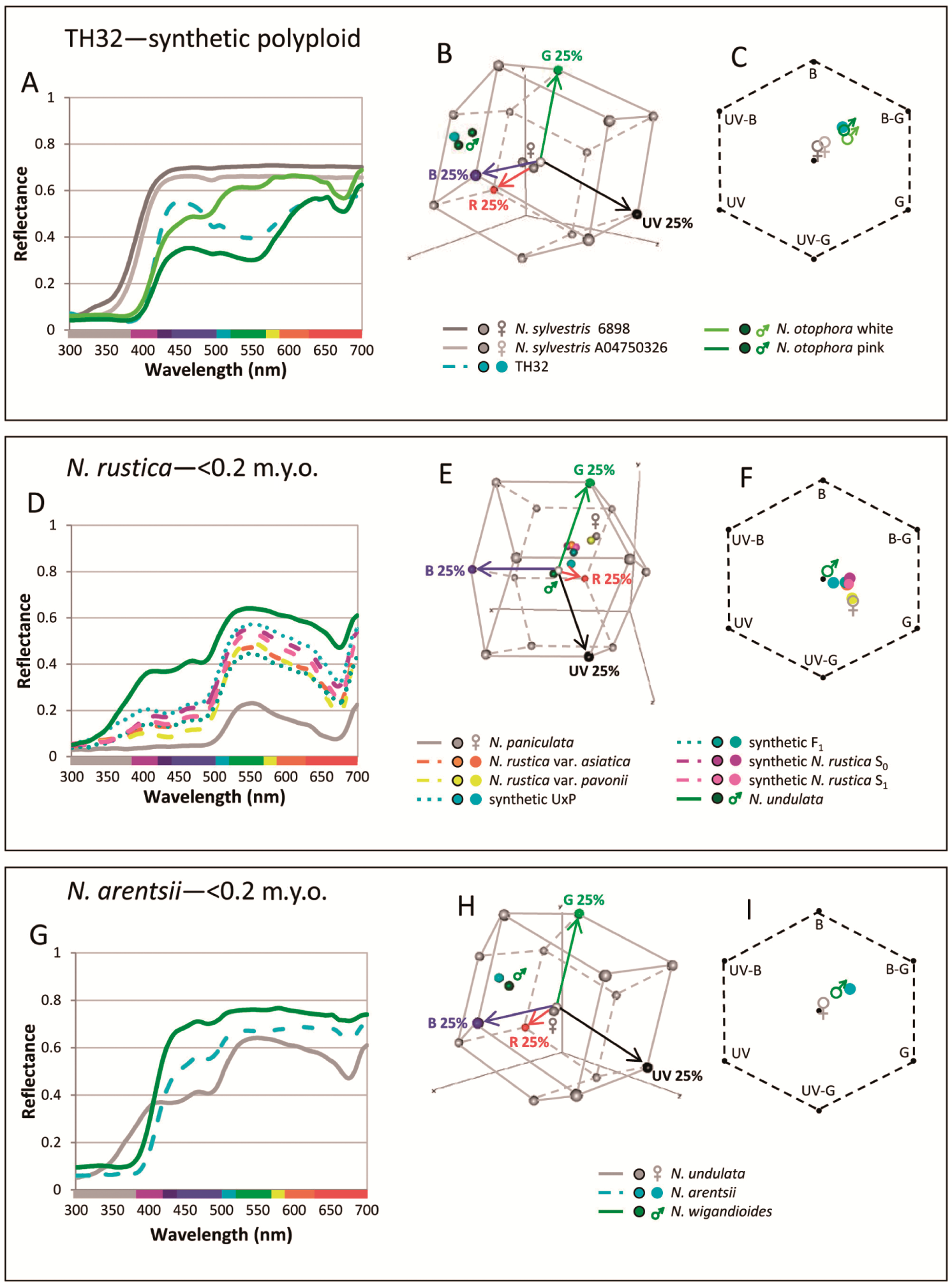
**

**Fig. S5** (A,D,G) Reflectance spectra for polyploid or homoploid sections and their progenitors (A) section *Polydicliae*, (D) section *Suaveolentes* (G) *N. glutinosa*. Solid lines are used for diploid taxa, dashed lines for polyploid taxa, and dotted lines for homoploid hybrid taxa. (B,E,H) Hummingbird colour space for polyploid or homoploid sections and their progenitors: (B) section *Polydicliae*, (E) section *Suaveolentes*, (H) *N. glutinosa*. The vertices of the hummingbird colour space represent 25% excitation of the photoreceptors; single photoreceptor type vertices (red, green, blue and UV) are coloured red, green, blue and black, respectively and all other vertices are grey. Red, green, blue and black arrows represent the vectors of these photoreceptors from the origin of the hummingbird colour space. (C,F,I) Bee colour hexagons for polyploid or homoploid sections and their progenitors: (C) section *Polydicliae*, (F) section *Suaveolentes*, (I) *N. glutinosa*. Hexagons have been scaled so that vertices represent 40% excitation of photoreceptors. UV=ultraviolet; UV-B=UV-blue; B=blue; B-G=blue-green; G=green; UV-G=UV-green. For information regarding how to interpret colour hexagons, see Supplemental Fig. S1. Female (♀) and male (♂) symbols mark maternal and paternal progenitors, respectively, in the hummingbird and bee colour spaces.

**
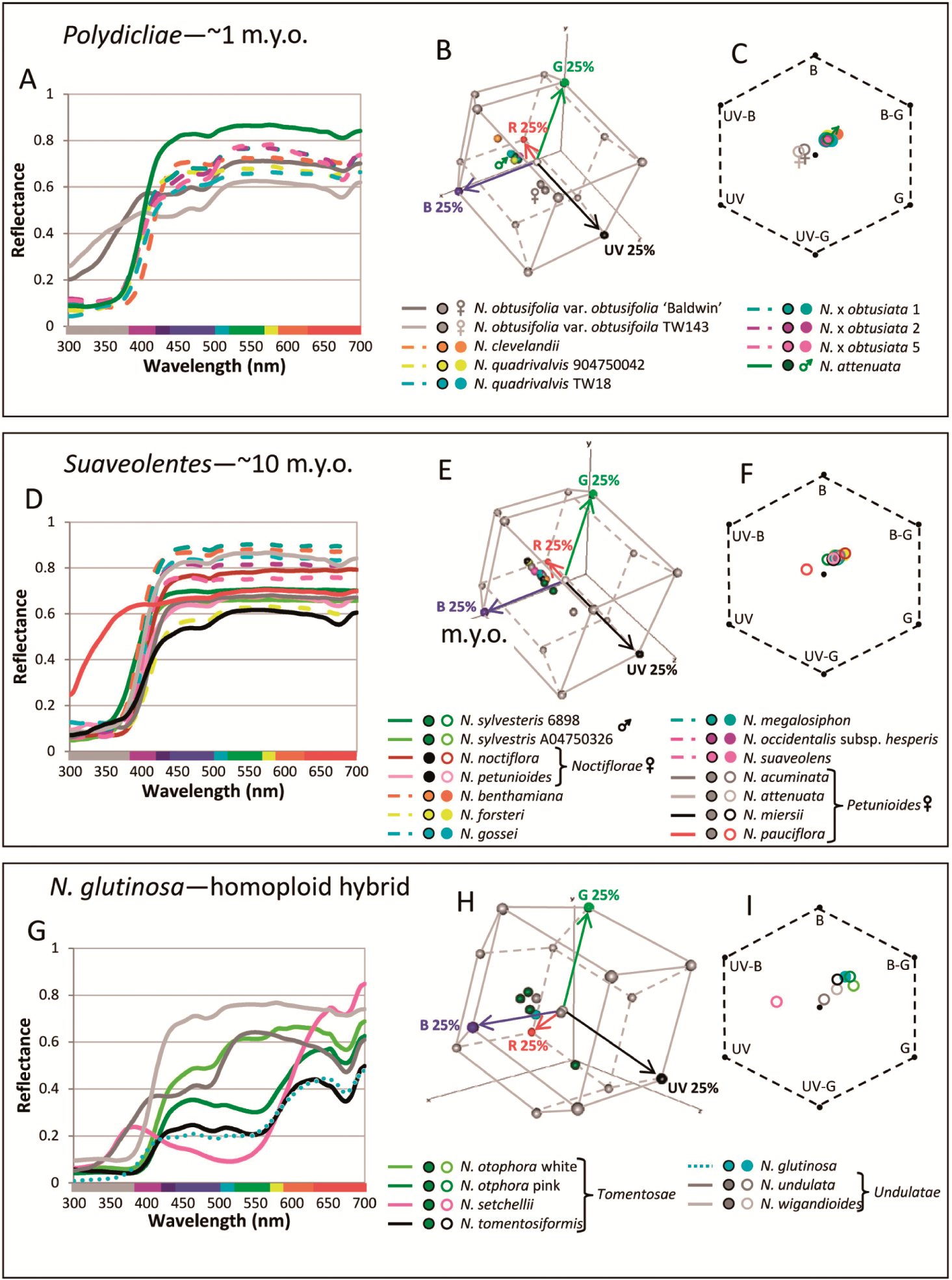
**

**Fig. S6** Results of the ancestral state reconstruction of the presence or absence of chlorophyll in corolla tissue summarised on the 95% majority rule tree from the Bayesian analysis of plastid sequence data from non-hybrid diploids. Homoploid and polyploid hybrids are superimposed on the diploid tree; black and grey solid, dashed and dotted lines to the right of the tree represent hybridisation events. Orange branches were added to the tree where progenitors of the hybrid taxa are entire sections. Pie charts at internal nodes indicate character states inferred for that node during ancestral state reconstruction carried out on a set of 36,000 post burn-in trees from the Bayesian analyses. Pie charts at the tips of the branches indicate character states observed in extant species.

**
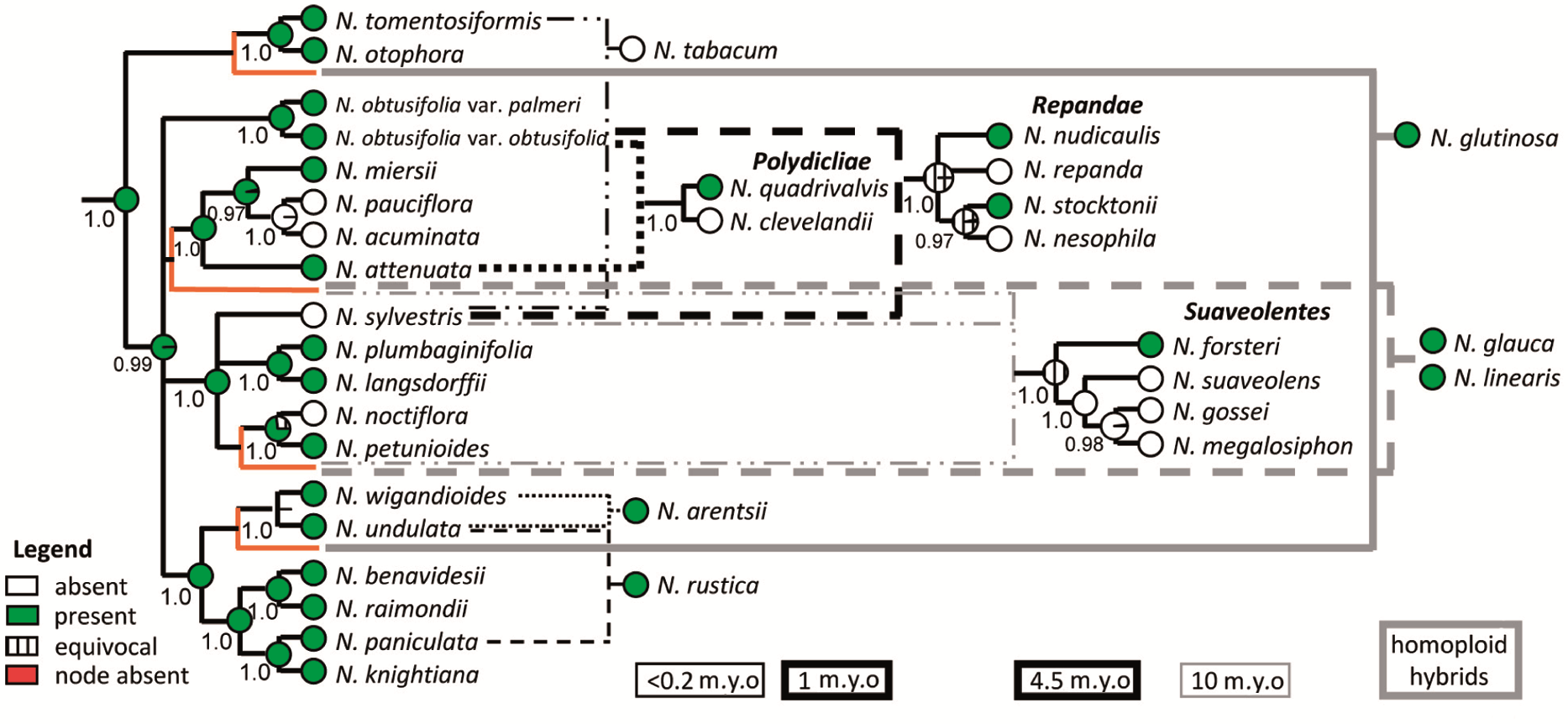
**

**Video.** Animation of *Nicotiana* spectra in 3D hummingbird colour space. *Nicotiana* loci are labelled according to hummingbird colour categories as in Fig. 4D: white=white; grey=UV-white; magenta=pink; yellow=synthetic *N. tabacum* QM; dark magenta=UV-pink; dark cyan= *N. tabacum* 095-55; red=*N. setchellii*; green=green; cyan=*N. langsdorffii*; dark green=saturated green; orange=UV-green. Hummingbird colour space vertices are represented at 50% excitation, and single photoreceptor types (red, green, blue and UV) are red, green, blue and black, respectively. All other vertices are grey. The origin is black.

**Literature Cited**

**Anssour S, Krugel T, Sharbel TF, Saluz HP, Bonaventure G, Baldwin IT.** **2009**. Phenotypic, genetic and genomic consequences of natural and synthetic polyploidization of *Nicotiana attenuata* and *Nicotiana obtusifolia*. *Annals of Botany,* **103**: 1207-1217.

**Burk LG.** **1973**. Partial self-fertility in a theoretical amphiploid progenitor of *N. tabacum*. *Journal of Heredity,* **64**: 348-350.

**Clarkson JJ.** **2006**. *Nicotiana (Solanaceae): Insights from molecular phylogenetics and cytogenetics,* PhD Thesis, Queen Mary, University of London, London, UK.

**Clarkson JJ, Lim KY, Kovarik A, Chase MW, Knapp S, Leitch AR.** **2005**. Long-term genome diploidization in allopolyploid *Nicotiana* section *Repandae* (Solanaceae). *New Phytologist,* **168**: 241-252.

**Leitch IJ, Hanson L, Lim KY*, et al.*** **2008**. The ups and downs of genome size evolution in polyploid species of *Nicotiana* (Solanaceae). *Annals of Botany,* **101**: 805-814.

**Moon HS, Nicholson JS, Lewis RS.** **2008**. Use of transferable *Nicotiana tabacum* L. microsatellite markers for investigating genetic diversity in the genus *Nicotiana*. *Genome,* **51**: 547-559.
